# Domino Reaction for the Sustainable Functionalization of Few-Layer Graphene

**DOI:** 10.3390/nano9010044

**Published:** 2018-12-30

**Authors:** Vincenzina Barbera, Luigi Brambilla, Alberto Milani, Alberto Palazzolo, Chiara Castiglioni, Alessandra Vitale, Roberta Bongiovanni, Maurizio Galimberti

**Affiliations:** 1 Politecnico di Milano, Department of Chemistry, Materials and Chemical Engineering “G. Natta”, piazza Leonardo da Vinci, 32-via Mancinelli 7, 20131 Milano, Italy; alberto.milani@polimi.it (A.M.); alberto.palazzolo@cea.fr (A.P.); chiara.castiglioni@polimi.it (C.C.); 2 Politecnico di Torino, Department of Applied Science and Technology, Corso Duca degli Abruzzi 24, 10129 Torino, Italy; alessandra.vitale@polito.it (A.V.); roberta.bongiovanni@polito.it (R.B.)

**Keywords:** graphene layers, pyrrole compounds, infrared spectroscopy, quantum chemical modelling, Density Functional Theory, Functional Groups

## Abstract

The mechanism for the functionalization of graphene layers with pyrrole compounds was investigated. Liquid 1,2,5-trimethylpyrrole (TMP) was heated in air in the presence of a high surface area nanosized graphite (HSAG), at temperatures between 80 °C and 180 °C. After the thermal treatments solid and liquid samples, separated by centrifugation, were analysed by means of Raman, Fourier Transform Infrared (FT-IR) spectroscopy, X-Rays Photoelectron Spectroscopy (XPS) and ^1^H-Nuclear Magnetic Resonance (^1^H NMR) spectroscopy and High Resolution Transmission Electron Microscopy (HRTEM). FT-IR spectra were interpreted with the support of Density Functional Theory (DFT) quantum chemical modelling. Raman findings suggested that the bulk structure of HSAG remained substantially unaltered, without intercalation products. FT-IR and XPS spectra showed the presence of oxidized TMP derivatives on the solid adducts, in a much larger amount than in the liquid. For thermal treatments at T ≥ 150 °C, IR spectral features revealed not only the presence of oxidized products but also the reaction of intra-annular double bond of TMP with HSAG. XPS spectroscopy showed the increase of the ratio between C(sp^2^)N bonds involved in the aromatic system and C(sp^3^)N bonds, resulting from reaction of the pyrrole moiety, observed while increasing the temperature from 130 °C to 180 °C. All these findings, supported by modeling, led to hypothesize a cascade reaction involving a carbocatalyzed oxidation of the pyrrole compound followed by Diels-Alder cycloaddition. Graphene layers play a twofold role: at the early stages of the reaction, they behave as a catalyst for the oxidation of TMP and then they become the substrate for the cycloaddition reaction. Such sustainable functionalization, which does not produce by-products, allows us to use the pyrrole compounds for decorating sp^2^ carbon allotropes without altering their bulk structure and smooths the path for their wider application.

## 1. Introduction

A large number of applications can be envisaged for graphene [1,2,3,4,5], because of its fascinating properties: high charge-carrier mobility [6,7,8], in-plane thermal conductivity [9,10,11], and elastic modulus of the order of 1 TPa [12,13,14]. Research performed over the last years has shown that application of carbon nanomaterials has to be assisted by functionalization. Thanks to functionalization, applications can be studied for energy [15], aerospace [16], in the biotechnological field [17,18], for cancer treatment [19,20], and drug transportation in biological systems [21,22,23,24]. Functionalization of graphene, preserving its bulk structure and controlling size, shape, and edge structure of the layers is indeed a challenging task [25,26,27,28,29,30,31,32,33,34,35,36,37,38,39,40,41,42].

In past works, some of the authors have reported on the functionalization of graphene layers with 2-(2,5-dimethyl-1*H*-pyrrol-1-yl)-1,3-propanediol (serinol pyrrole, SP), a serinol derivative prepared through the neat reaction of 2-amino-1,3-propandiol with 2,5-hexanedione, with atomic efficiency up to about 85% [43]. Functionalization occurred in the absence of solvents and catalysts, by simply providing either thermal or mechanical energy; functionalization yield was almost quantitative, however it was larger than 90%. Functionalization was indeed sustainable. Pyrrole adducts could be even formed on graphite substrates. Very stable interaction between the carbon allotropes and SP or SP derivatives was documented and stacks of few layers graphene revealed an unaltered interlayer distance and bulk crystalline structure [43,44,45]. Functionalization appeared to occur on peripheral positions, mostly on the edges of graphene layers. Functionalization of graphene layers has also been performed with pyrrole compounds (PyC) bearing different substituents on the nitrogen atom [46], again without appreciably altering the structure of the graphene layers and without observing expansion of their interlayer distance. Functionalization yield was up to about 90%. The addition of PyC, was shown as a simple and effective way to modify the Hansen solubility parameters of the graphene layers [46]. Adducts of PyC have also been prepared with carbon allotropes (CA) other than nanosized graphites, such as carbon nanotubes (CNT) and furnace carbon black [44,45,46,47,48], obtaining functionalization yields larger than 80% and almost quantitative in the case of high surface area CNT. Applications of carbon allotropes adducts with PyC have been reported, from the preparation of conductive inks to polymer composites [44,45,46,47,48].

In the light of these results, it seemed worthwhile to pursue the understanding of PyC adducts with graphene layers, focusing the attention on the nature of the interaction, in particular on the mechanism which leads to the formation of the adducts.

**Figure 1:**
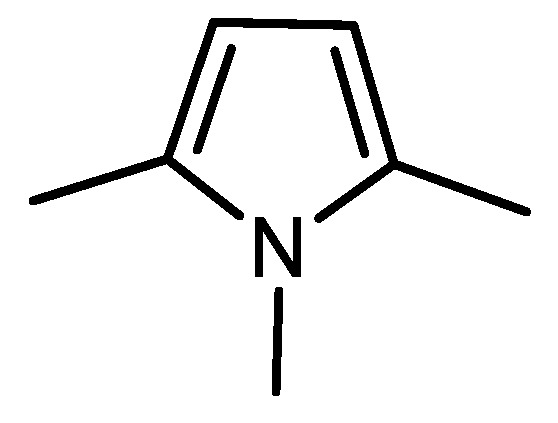
Structure formula of 1,2,5-trimethylpyrrole (TMP).

A pyrrole compound was selected as model system: 1,2,5-trimethylpyrrole (trimethylpyrrole, TMP) (Figure 1).

The interaction of TMP was studied with a nanosized graphite with high surface area and high shape anisotropy (HSAG) [49], that means a high crystalline order inside the plane and a low number of layers stacked in crystalline domains. HSAG has been used in previous studies [43,46,47,48,49,50,51].

TMP and the TMP/HSAG mixtures were kept in air at 80, 100, 130 and 150 °C for 120 min, in the absence of solvents and catalysts. HSAG/TMP molar ratio was <<1 or ≈1, by estimating the molar amount of HSAG through the moles of graphene structural units (C_2_). Spectroscopic investigations were performed by means of Raman Spectroscopy, Fourier Transform Infrared Spectroscopy (FT-IR) and X-Ray Photoelectron Spectroscopy (XPS), on TMP and TMP/HSAG, treated at the reported temperatures. Quantum chemical modelling of the IR spectra was done by means of Density Functional Theory (DFT) calculations of several model molecules. A combined experimental and computational approach was thus applied, which had been proved to be very powerful for the accurate interpretation of the spectra of complex molecular systems and materials [52,53,54].

## 2. Experimental Part

### 2.1. Materials and Syntheses

Reagents and solvents were commercially available and were used without further purification: 1,2,5-trimethylpyrrole, methylamine hydrochloride, acetone, 2,5-hexanedione deuterated chloroform (CDCl_3_) and dimethylsulfoxide (DMSO-*d*6) were from Sigma-Aldrich. Synthetic Graphite 8427^®^ (Asbury Graphite Mills Inc., Asbury, NJ, USA), indicated in the text as high surface area graphite (HSAG).

#### 2.1.1. High Surface Area Graphite

High surface area graphite (HSAG) was Synthetic Graphite 8427^®^ (Asbury Graphite Mills Inc.). Characterization of HSAG has been reported by some of the authors in previous works [43,46,47,48,49,50,51]. Chemical composition of HSAG, determined by elemental analysis, was (mass %): carbon 99.5, hydrogen 0.4, nitrogen 0.1, oxygen 0.0. TGA revealed the following mass loss: 3.2% below 700 °C. Surface area was determined by BET according to ASTM D6556 method and was found to be 330.3 m^2^/g. Average size of HSAG particles was evaluated by means of dynamic light scattering [43,50], obtaining values representing the hydrodynamic radius of HSAG particles in water dispersions. Average values were 500 nm in the as prepared dispersion and 190 nm after centrifuging the dispersion for 30 min centrifugation at 9000 rpm. Transmission electron micrograph taken on supernatant suspension after 60 min centrifugation at 9000 rpm revealed graphite stacks randomly arranged, with lateral size between about 300 nm and 500 nm [50].

#### 2.1.2. Synthesis of 1,2,5-Trimethylpyrrole

1 g of methylamine hydrochloride (14.8 mmol) and 1.69 g of 2,5-hexanedione (14.8 mmol) were poured in a 100 mL round bottom flask equipped with magnetic stirrer and condenser. The mixture was left to stir at 150 °C for 2 hours. After this time the reaction mixture was cooled to room temperature. Pure product was obtained yielding 1.4 g (86.9%). ^1^H NMR (CDCl_3_, 400 MHz); δ (ppm) = 5.77 (s, 2H, =CH), 3.39 (s, 3H, N–CH_3_), 2.21 (s, 6H, CH_3_). ^13^C NMR (CDCl_3_, 100 MHz); δ (ppm) = 128, 106.37, 106.00, 32.01, 13.22.

#### 2.1.3. Synthesis of 1,2,5-Trimethylpyrrole on HSAG as Support

1 g of methylamine hydrochloride (14.8 mmol), 1.69 g of 2,5-hexanedione (14.8 mmol) and HSAG (1.06 g) were poured in a 100 mL round bottom flask equipped with magnetic stirrer and condenser. The mixture was left to stir at 150 °C for 2 hours. After this time the reaction mixture was cooled to room temperature. The black mixture was placed in a Büchner funnel with a sintered glass disc, washed with acetone (15 mL). Pure product was obtained after removing of solvent and yielding 1.1 g (68.3%). ^1^H NMR (CDCl_3_, 400 MHz); δ (ppm) = 5.77 (s, 2H, =CH), 3.39 (s, 3H, N–CH_3_), 2.21 (s, 6H, CH_3_). ^13^C NMR (CDCl_3_, 100 MHz); δ (ppm) = 128, 106.37, 106.00, 32.01, 13.22.

#### 2.1.4. Thermal Treatments of 1,2,5-Trimethylpyrrole at Different Temperatures: 80 °C, 100 °C, 130 °C, 150 °C and 180 °C

##### General Procedure

In a 10 mL glass vial equipped with magnetical stirrer was put 1,2,5-trimethylpyrrole (100 mg, 0.91 mmol). The oil was heated at the selected temperature for 2 h. After this time, the mixture was kept at room temperature and analyzed by means of NMR and FT-IR spectroscopies.

##### TMP at 80 °C and 100 °C. TMP

^1^H NMR (CDCl_3_, 400 MHz); δ (ppm) = 5.77 (s, 2H, TMP), 5.74 (d, 2H, (DMP)_2_), 5.62 (d, 2H, (DMP)_2_), 3.65 (s, 6H, (DMP)_2_), 3.39 (s, 3H, TMP), 2.21 (s, 6H, TMP), 2.14 (s, 6H, (DMP)_2_).

##### TMP at 130 °C and 150 °C. TMP and Traces of DMP-CHO

^1^H NMR (CDCl_3_, 400 MHz); δ (ppm) = 9.4 (s, 1H, DMP-CHO), 6.82 (d, 1H, DMP-CHO), 6.01 (d, 1H, DMP-CHO), 5.77 (s, 2H, TMP), 3.83 (s, 3H, DMP-CHO), 3.39 (s, 3H, TMP), 2.47 (s, 3H, DMP-CHO), 2.21 (s, 6H, TMP).

##### TMP at 180 °C. Mixture of DMP-CHO/MP-(CHO)_2_/TMP (1/1/50)

^1^H NMR (CDCl_3_, 400 MHz); δ (ppm) = 9.8 (s, 2H, MP-(CHO)_2_), 9.4 (s, 1H, DMP-CHO), 6.82 (d, 1H, DMP-CHO), 6.01 (d, 1H, DMP-CHO), 6.27 (s, 2H, MP-(CHO)_2_), 5.77 (s, 2H, TMP), 3.9 (s, 3H, MP-(CHO)_2_), 3.83 (s, 3H, DMP-CHO), 3.39 (s, 3H, TMP), 2.47 (s, 3H, DMP-CHO), 2.21 (s, 6H, TMP).

#### 2.1.5. Thermal Treatments of 1,2,5-Trimethylpyrrole Using a Catalytic Amount of HSAG at Different Temperatures: 80 °C, 100 °C, 130 °C, 150 °C and 180 °C

##### General Procedure

In a 50 mL round bottom flask equipped with magnetic stirrer were put HSAG (10 mg, 0.13 mmol) and TMP (1 g, 9.16 mmol) in sequence. The resulting mixture was heated at the selected temperature for 2 h. After this time the mixture was transferred into a Falcon™ tube (15 mL) and acetone (10 mL) was added. The suspension was sonicated for 10 min and centrifuged at 9000 rpm for 60 min (3 times).

The solid was analyzed by means of FT-IR spectroscopy.

The liquid was transferred in a round bottom flask, the solvent was removed under reduced pressure, the residue was analyzed by means of FT-IR and NMR spectroscopies.

##### HSAG-TMP(cat) at 80 °C. Mixture of (DMP)_2_/TMP (1/10)

^1^H NMR (CDCl_3_, 400 MHz); δ (ppm)= 5.77 (s, 2H, TMP), 5.74 (d, 2H, (DMP)_2_), 5.62 (d, 2H, (DMP)_2_), 3.65 (s, 6H, (DMP)_2_), 3.39 (s, 3H, TMP), 2.21 (s, 6H, TMP), 2.14 (s, 6H, (DMP)_2_).

##### HSAG-TMP(cat) at 100 °C. Mixture of MP-(CHO)_2_/(DMP)_2_/TMP (1/15/3)

^1^H NMR (CDCl_3_, 400 MHz); δ (ppm) = 9.8 (s, 2H, MP-(CHO)_2_), 6.27 (s, 2H, MP-(CHO)_2_), 5.77 (s, 2H, TMP), 5.74 (d, 2H, (DMP)_2_), 5.62 (d, 2H, (DMP)_2_), 3.9 (s, 3H, MP-(CHO)_2_), 3.65 (s, 6H, (DMP)_2_), 3.39 (s, 3H, TMP), 2.21 (s, 6H, TMP), 2.14 (s, 6H, (DMP)_2_).

##### HSAG-TMP(cat) at 130 °C. Amide-CHO and Traces of Oligopyrroles

^1^H NMR (CDCl_3_, 400 MHz); δ (ppm)= 9.7 (s, 1H, Amide-CHO), 6.05 (s, 1H, Amide-CHO), 2.22 (s, 3H, Amide-CHO).

##### HSAG-TMP(cat) at 150°C. Mixture of DMP-CHO/MP-(CHO)_2_/(DMP)_2_/TMP (1/1/8/5)

^1^H NMR (CDCl_3_, 400 MHz); δ (ppm) = 9.8 (s, 2H, MP-(CHO)_2_), 9.4 (s, 1H, DMP-CHO), 6.82 (d, 1H, DMP-CHO), 6.01 (d, 1H, DMP-CHO), 6.27 (s, 2H, MP-(CHO)_2_), 5.77 (s, 2H, TMP), 5.74 (d, 2H, (DMP)_2_), 5.62 (d, 2H, (DMP)_2_), 3.9 (s, 3H, MP-(CHO)_2_), 3.83 (s, 3H, DMP-CHO), 3.65 (s, 6H, (DMP)_2_), 3.39 (s, 3H, TMP), 2.47 (s, 3H, DMP-CHO), 2.21 (s, 6H, TMP), 2.14 (s, 6H, (DMP)_2_).

##### HSAG-TMP(cat) at 180 °C. Mixture of DMP-CHO/MP-(CHO)_2_/(DMP)_2_/TMP (1/1/30/5)

^1^H NMR (CDCl_3_, 400 MHz); δ (ppm) = 9.8 (s, 2H, MP-(CHO)_2_), 9.4 (s, 1H, DMP-CHO), 6.82 (d, 1H, DMP-CHO), 6.01 (d, 1H, DMP-CHO), 6.27 (s, 2H, MP-(CHO)_2_), 5.77 (s, 2H, TMP), 5.74 (d, 2H, (DMP)_2_), 5.62 (d, 2H, (DMP)_2_), 3.9 (s, 3H, MP-(CHO)_2_), 3.83 (s, 3H, DMP-CHO), 3.65 (s, 6H, (DMP)_2_), 3.39 (s, 3H, TMP), 2.47 (s, 3H, DMP-CHO), 2.21 (s, 6H, TMP), 2.14 (s, 6H, (DMP)_2_).

#### 2.1.6. Synthesis of HSAG-TMP Adducts (1:1 as Molar Ratio): 80 °C, 100 °C, 130 °C, 150 °C and 180 °C

##### General Procedure

In a 50 mL round bottom flask equipped with magnetic stirrer were put HSAG (0.5 g, 6.9 mmol) and TMP (0.75 g, 6.9 mmol) in sequence. The resulting mixture was heated at the selected temperature for 2 h. After this time the solid was transferred into a Falcon™ tube (15 mL) and acetone (10 mL) was added. The suspension was sonicated for 10 min and centrifuged at 9000 rpm for 60 min (3 times).

The same procedure was adopted for the preparations of all the adducts presented in this manuscript. Adducts were obtained as a black powder. Thermogravimetric analysis was performed after each functionalization (Figure SI-1). Functionalization yield was determined through the following equation (Equation 1):

(1) $$\mathrm{Functionalization}\;\mathrm{Yield}\;(\%)\;=\;100\;*\;\frac{\mathrm{PyC}\;\mathrm{mass}\;\%\;\mathrm{in}\;(\mathrm{HSAG}-\mathrm{PyC}\;\mathrm{adduct})\;\mathrm{after}\;\mathrm{acetone}\;\mathrm{washing}}{\mathrm{PyC}\;\mathrm{mass}\;\%\;\mathrm{in}\;(\mathrm{HSAG}-\mathrm{PyC}\;\mathrm{adduct})\;\mathrm{before}\;\mathrm{acetone}\;\mathrm{washing}}$$

% mass of TMP in HSAG-TMP adduct were obtained by weighting after removing the solvent at reduced pressure.

HSAG-TMP(1/1) at 80°C. Functionalization yield: 12 %.
HSAG-TMP(1/1) at 100°C. Functionalization yield: 20 %.
HSAG-TMP(1/1) at 130°C. Functionalization yield: 42 %.
HSAG-TMP(1/1) at 150°C. Functionalization yield: 56 %.
HSAG-TMP(1/1) at 180°C. Functionalization yield: 55 %.

### 2.2. Characterization of HSAG and Reaction Products

#### 2.2.1. Fourier Transform Infra Red spectroscopy (FT-IR)

The IR spectra were recorded in transmission mode (128 scan and 4 cm^−1^ resolution) using a ThermoElectron Continuμm IR microscope coupled with a FTIR Nicolet Nexus spectrometer. For the cases of the liquid samples a small drop was casted on ZnSe windows and analyzed in transmission mode. For solid samples a small portion of the material was placed in a diamond anvil cell (DAC) and analyzed in transmission mode.

#### 2.2.2. Raman Spectroscopy

Raman spectra of powder samples deposited on a glass slide were recorded with a Horiba Jobin Yvon Labram HR800 dispersive Raman spectrometer equipped with Olympus BX41 microscope and a 50 × objective. The excitation line at 632.8 nm of a He/Ne laser was kept at 0.5 mW in order to prevent samples degradation. The spectra were obtained as the average of four acquisitions (30 s for each acquisition) with a spectral resolution of 2 cm^−1^.

#### 2.2.3. High Resolution X-ray Photoelectron Spectroscopy (XPS)

PHI 5000 VersaProbe instrument (Physical Electronics) was utilized for survey scan and high-resolution (HR) XPS. The powder was placed in the XPS pre-chamber overnight, in order to avoid anomalous outgassing during the XPS characterization, performed in UHV conditions (10^−8^ Pa). A monochromatic Al K-alpha X-ray source (1486.6 eV energy, 15 kV voltage and 1 mA anode current) and a power of 25.2 W were used for analysis. Different pass energy values were exploited: 187.85 eV for survey spectra and 23.5 eV for high-resolution peaks. Analyses were carried out with a take-off angle of 45° and with a 100 μm diameter X-ray spot size. A double beam (electron and argon ion gun) neutralization system, dedicated to reduce the charging effect on samples, was also employed during data acquisition. Spectra were analyzed and peak deconvolution was performed using Multipak 9.6 software. All core-level binding energies were referenced to the C1s line at 284.5 eV and the background contribution in HR scans was subtracted by means of a Shirley function.

#### 2.2.4. High-Resolution Transmission Electron Microscopy (HRTEM)

HRTEM investigations on HSAG and HSAG/TMP samples taken from the sonicated suspensions, were carried out with a Philips CM 200 field emission gun microscope operating at an accelerating voltage of 200 kV. Few drops of the water suspensions were deposited on 200 mesh lacey carbon-coated copper grid and air-dried for several hours before analysis. During acquisition of HRTEM images, the samples did not undergo structural transformation. Low beam current densities and short acquisition times were adopted. To estimate the number of stacked graphene layers and the dimensions of the stacks visible in HRTEM micrographs, the Gatan Digital Micrograph software was used.

#### 2.2.5. Quantum Chemical Molecular Modeling

The interpretation of the experimental spectra have been supported through quantum chemical molecular modeling: based on the models reported in Table 2, geometry optimization and IR spectra calculation have been computed in the framework of Density Functional Theory (DFT) by using Gaussian09 package [55] Hybrid B3LYP exchange-correlation functional has been employed together with 6-311++G(d,p) basis set. A frequency scaling factor of 0.978 have been applied to all the computed spectra reported in this paper: this factor has been determined by using TMP as a reference case and in particular it is the scaling factor required to put the reference band computed at 1042 cm^−1^ in correspondence to the band experimentally observed at 1019 cm^−1^.

## 3. Results and Discussion

### 3.1 Thermal Treatment of TMP

In a first series of experiments, a TMP sample, whose ^1^H NMR spectrum did not reveal any impurity (Supplementary Information), was split in different portions, which were heated in air at 25 °C, 80 °C, 100 °C, 130 °C, 150 °C and 180 °C, both in the absence and in the presence of a catalytic amount of HSAG. The HSAG/TMP molar ratio was about 1:100, estimating the molar amount of HSAG with respect to the moles of graphene structural units (C_2_). After the thermal treatment, liquid substances were isolated and were characterized by means of solution ^1^H NMR and FT-IR carried out in transmission, focusing the IR beam on a thin layer obtained from drop casting the liquid sample on a ZnSe window. ^1^H NMR analyses were performed in liquid phase immediately after thermal treatments. IR spectra of a few scans were collected immediately after drop deposition (spectra recorded before a substantial evaporation of the volatile fraction). Other spectra were recorded about two minutes after drop deposition (after evaporation of volatile molecules). Theoretical IR spectra were generated by means of DFT calculations (B3LYP/6-311++G(d,p)) *in vacuo*, both for TMP and for model molecules derivatives of TMP which were hypothesized to arise from the thermal treatments. Assignments of spectral features and thus determination of the molecular structure were performed by cross-examining experimental findings and theoretical simulations.

#### 3.1.1 Thermal Treatment of TMP in the Absence of HSAG

**Figure 2:**
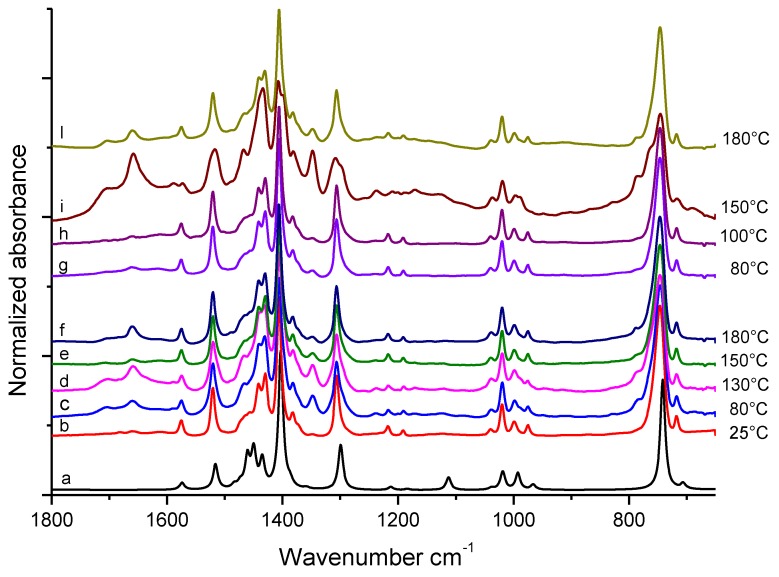
FT-IR spectra, in the 1800–700 cm^−1^ region, of TMP samples heated in air at different temperatures. On the right side of the Figure, treatment temperatures (°C) are indicated. (**a**): theoretical DFT spectrum of TMP. (**b**): pristine TMP; (**c**)–(**f**): samples treated in the absence of HSAG. (**g**)–(**l**): samples treated in the presence of catalytic amount of HSAG. All the spectra were obtained immediately after drop deposition on ZnSe window, from the collection of the first few scans.

FT-IR spectra of TMP based samples, (pristine TMP and pristine TMP treated at different temperatures), recorded before substantial evaporation of the volatile fraction, are shown in Figure 2. Spectrum (b) is of pristine TMP (stored at 25 °C) and spectra (c), (d), (e) and (f) were taken on TMP treated at: 80 °C, 130 °C, 150 °C, and 180 °C, respectively. At the bottom of Figure 2 the theoretical spectrum (a) of TMP, obtained by means of DFT calculations (B3LYP/6-311++G(d,p)) *in vacuo*, is shown for comparison.

The good correspondence between DFT spectrum (a) and the spectrum of pristine TMP (b) proves that the adopted theory is adequate and can be exploited for the assignment of IR absorption features by means of vibrational eigenvectors analysis. The proposed assignment of the main peaks of spectrum (b) of Figure 2 is reported in Table 1, which shows the comparison among experimental and theoretical vibrational wavenumbers.

Spectra from (b) to (f) in Figure 2 are dominated by the features of pristine TMP, independently of the treatment temperature (from 25 °C to 180 °C). However, weak absorptions, which cannot be attributed to TMP, are present in the spectra of samples treated at temperatures ≥ 80 °C, particularly in the 1800–1600 cm^−1^ region.

^1^H NMR spectra of TMP samples heated in the absence of HSAG are provided as Supplementary Information. Figure SI-2 shows the ^1^H NMR spectra of samples from treatments at 80 °C, 100 °C, 130 °C, 150 °C, and 180 °C. In all the spectra, signals due to TMP are predominant. Spectra of samples treated at temperatures ≥ 130 °C reveal low intensity signals due to species (almost traces) other than TMP.

#### 3.1.2 Thermal Treatment of TMP in the Presence of a Catalytic Amount of HSAG

After the thermal treatments, solid samples were rinsed with acetone until the washing solvent did not reveal the presence of any organic substance (details are in the experimental part) and were then isolated. Figure 2 reports also the FT-IR spectra of TMP samples treated at 80 °C, 100 °C, 150 °C, and 180 °C respectively (spectra from (g) to (l)), in the presence of catalytic amount of HSAG. Features of pristine TMP dominate the spectra of samples treated at 80 °C and 100 °C. The same new features already observed as weak absorption for samples treated at the higher temperature in the absence of HSAG (spectra (d)–(f)), now appear as medium-strong bands in the spectrum (i) of the sample treated at 150 °C and are still evident in spectrum (l) (180 °C, with HSAG). Moreover, the sample treated at 130 °C in the presence of HSAG shows a remarkably different IR spectrum (not reported in Figure 2): this spectrum shows the presence of amidic species, as discussed in more detail below.

^1^H NMR spectra of samples treated in the presence of catalytic amount of HSAG are shown in Figure SI-3. Peaks due to TMP are present in all the spectra except for that of the sample treated at 130 °C. In accordance with the indication from IR analysis, additional signals due to species other than TMP can be detected in the ^1^H NMR spectra of samples treated at temperature ≥ 80 °C and they change their relative intensity as the heating temperature increases. Besides the absence of peaks due to TMP, the ^1^H NMR spectrum of the sample treated at 130 °C shows features which are not present in the other spectra.

**Figure 3:**
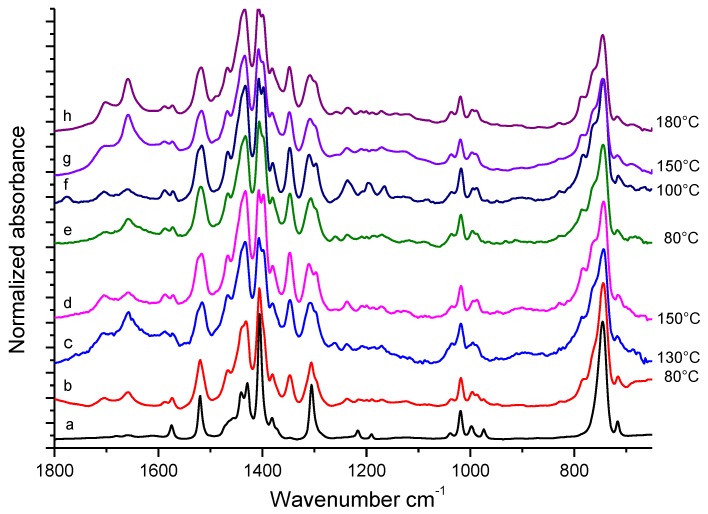
Experimental FT-IR spectra, in the 1800–700 cm^−1^ region, of TMP samples heated in air at different temperatures. Spectra were recorded two minutes after drop deposition. On the right side of the Figure, treatment temperatures (°C) are indicated. (**a**): pristine TMP. (**b**–**d**): samples treated in the absence of HSAG. (**e**–**h**): samples treated in the presence of catalytic amount of HSAG.

FT-IR spectra were then taken on the same samples, kept at different temperatures in the absence and in the presence of a catalytic amount of HSAG, about two minutes after drops’ deposition on ZnSe window. These spectra are shown in Figure 3, in comparison with the spectrum of pristine TMP (spectrum (a)).

In the spectra of samples treated at temperatures ≥ 80 °C, peaks due to TMP appear together with absorption features of medium-strong intensity, different from those of pure TMP. These bands appear for samples heated in the absence of HSAG (spectra (b)–(d) in Figure 3) or in the presence of a catalytic amount of HSAG (spectra (e)–(h) in Figure 3). Most of these new features were already observed as weak absorptions in the spectra of Figure 2, obtained immediately after drop deposition. This suggests that the absorption features not due to TMP belong to non-volatile chemical species formed as a consequence of the thermal treatment and whose relative amount increases after the evaporation of TMP. Even if the comparison among spectra in Figure 2 and Figure 3 seem to indicate that samples treated in the presence of HSAG present a higher concentration of species other than TMP, it is impossible to assess their relative amount in the different samples on the basis of only IR evidence.

Hypotheses were formulated for the structure of molecules arising from TMP heating, at temperatures ≥ 80 °C. A set of model molecules derivatives of TMP, were considered for DFT simulations of the IR spectrum. The comparative exam of the above reported experimental findings from FT-IR and ^1^H NMR, which in conjunction with DFT calculations led to identifying molecular structures compatible with the experimental data. Such structures are shown in Table 2.

**Figure 4:**
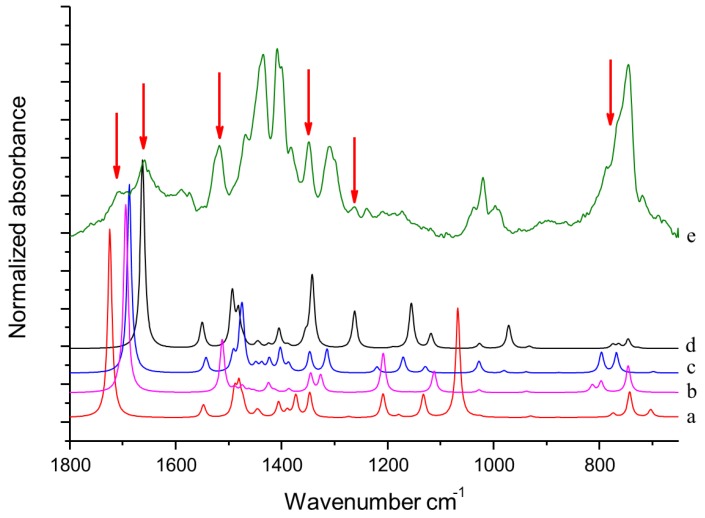
DFT computed spectra of: DMP-COOH (a), MP-(CHO)_2_ (b), DMP-CHO (c), and DMP-COOH H-bonded dimer (d). The experimental reference spectrum (e) of modified TMP (sample treated at 130 °C in the absence of HSAG, spectrum recorded after TMP evaporation) is reported, where red arrows indicate absorption features, which can be rationalized with the occurrence of the oxidized species.

DFT computed spectra of all the oxidized species reported in Table 1 are shown in Figure 4: 1,5-dimethyl-1*H*-pyrrole-2-carbaldehyde, DMP-COOH (**a**), 1-methyl-1*H*-pyrrole-2,5-dicarbaldehyde, MP-(CHO)_2_ (**b**), 1,5-dimethyl-1*H*-pyrrole-2-carboxylic acid, DMP-CHO (**c**), and DMP-COOH hydrogen bonded dimer (**d**).

In Figure 4 it is also shown for comparison the IR spectrum of TMP sample treated at 130 °C in the absence of HSAG (e). This spectrum has been recorded after the evaporation of TMP, and will be hereafter taken as the reference spectrum of chemically modified TMP. Indeed, it clearly shows all the absorption features ascribed to species arising from thermal treatment of TMP (bands are less or more strong, for the different samples), which were detected in all the spectra of TMP samples analyzed here.

The following comments can be made. The intensification and the broadening of the absorption band at about 1516 cm^−1^, observed in the spectra in Figure 2 and Figure 3, can be explained by the strong absorptions predicted for all the model structures in the region 1450–1550 cm^−1^. Bands assigned to C=O stretching vibration of different species (aldehydes and acids) can justify the wide and structured feature observed in the 1600–1750 cm^−1^ region. It is worth noting that the presence of hydrogen bonded acid species is essential for explaining the C=O stretching component lying at relatively low frequency (1657 cm^−1^). Both acids and aldehydes can justify the strong new feature at 1345 cm^−1^ and the remarkable broadening of the CH out-of-plane feature below 800 cm^−1^. A small yet clearly detectable absorption at 1260 cm^−1^ is nicely accounted for by the spectrum of the H bonded acid dimer. In Figure 4, the red arrows indicate absorption features which can be rationalized with the occurrence of DMP-CHO, MP-(CHO)_2_, DMP-COOH and DMP-COOH H-bonded dimer as the oxidized species, as discussed above.

**Figure 5:**
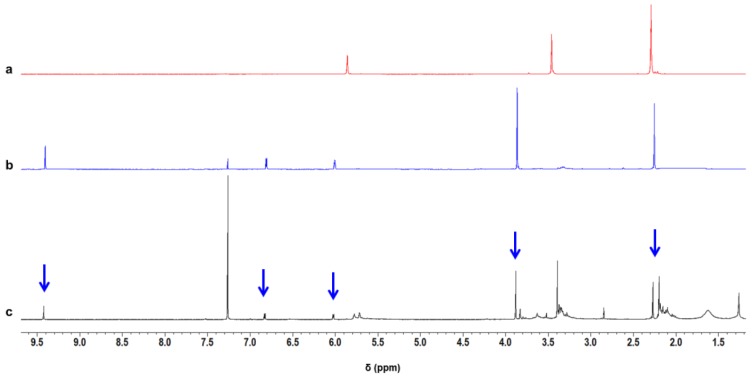
^1^H NMR spectra (400 MHz, CDCl_3_) of pristine TMP (**a**), DMP-CHO (see Table 2) (**b**), liquid product obtained by heating TMP in the presence of catalytic amount of HSAG at 150 °C (**c**). The arrows indicate the peaks assigned to DMP-CHO.

The IR analysis reveal that the proposed model compounds can account for several IR experimental findings and their identification was further validated by means of ^1^H NMR (Figures SI-2–SI-4) and Gas Chromatography–Mass Spectrometry (GC-MS) analysis (Supplementary Information Figure SI-5). DMP-CHO was isolated from the product mixture obtained by heating TMP at 100 °C in the presence of a catalytic amount of HSAG. ^1^H NMR spectrum of DMP-CHO is shown in Figure SI-4 and is compared in Figure 5 (spectrum b) with the spectra of pristine TMP (spectrum a) and of the liquid product obtained by heating TMP in the presence of catalytic amount of HSAG at 150 °C (spectrum c).

The comparison of DMP-CHO ^1^H NMR spectrum with the ^1^H NMR spectra of TMP samples treated at different temperatures (see also Figure SI-3) reveals that DMP-CHO was formed at 80 °C, 100 °C, 130 °C and 150 °C: only in traces in the absence of HSAG and in an appreciable amount in the presence of a catalytic amount of HSAG.

**Figure 6:**
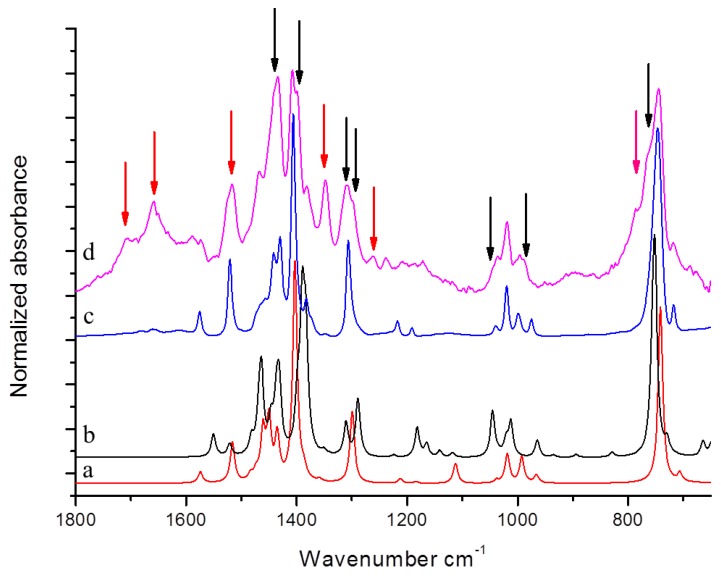
DFT computed spectrum of: TMP (**a**) and of α-α′ dimer of TMP (DMP)_2_ (**b**). Experimental spectra of pristine TMP (**c**) and the reference spectrum of modified TMP-sample treated at 130 °C in the absence of HSAG, spectrum recorded after TMP evaporation (**d**). Black and red arrows indicate absorption features which can be rationalized with the occurrence of (DMP)_2_ and TMP oxidized species respectively.

However, there are features in both IR and ^1^H NMR spectra which cannot be assigned to the molecular structures of TMP and oxidized TMP. They can be ascribed to the dimeric species 1,1′,5,5′-tetramethyl-1*H*,1′*H*-2,2′-bipyrrole, (DMP)_2_. In Figure 6, DFT computed spectrum of TMP (a) and (DMP)_2_ (b) are shown. These theoretical spectra are compared with the reference spectrum of modified TMP treated at 130 °C (d) and with the spectrum of pristine TMP (c).

By directly comparing spectra (c) and (d) in Figure 6, it is possible to clearly detect new features due to TMP derivatives formed by the thermal treatment.

It can be thus commented that the features labelled by red arrows in Figure 4 and Figure 6 are justified on the basis of oxidized species (as already discussed above), while DFT predictions for TMP and for (DMP)_2_ allow us to explain the absorption peaks indicated with black arrows in Figure 6. (DMP)_2 _species are indeed responsible for the appearance of a higher frequency components of the CH out of plane band (765–788 cm^−1^), of the band shape evolution of the group of bands around 1000 cm^−1^, and of the remarkable broadening of the band at about 1307 cm^−1^, which clearly results from the convolution of at least two components. Moreover, the presence of the three strong transitions in the C–C stretching region predicted for (DMP)_2_ could partially explain the raise in intensity of the structured band with maximum at 1433 cm^−1^ and the broadening of the band observed at 1405 cm^−1^.

Signals due to (DMP)_2_ can be detected in ^1^H NMR spectra of TMP samples treated, in the presence of catalytic amount of HSAG, at T ≥ 80 °C (Figure SI-3d,e) and in the absence of HSAG at 180 °C. As is shown in more detail as Supplementary Information, the relative amount of (DMP)_2_ with respect to TMP appears to dramatically increase at T = 100 °C. Interestingly, the amount of (DMP)_2_ is larger than the amount of oxidized TMP, at every temperature and also at 180 °C in the absence of HSAG.

**Figure 7:**
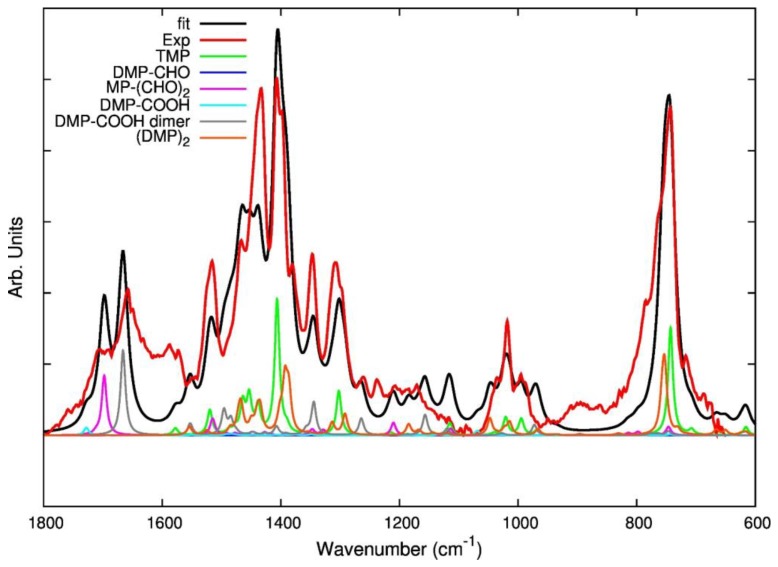
Result of the fitting of the experimental spectrum (red line) of a TMP sample after thermal treatment (reference spectrum of modified TMP (sample treated at 130 °C, spectrum recorded after TMP evaporation). The fitting curve (black line) is obtained as a weighted sum of theoretically predicted spectra for some model molecules (oxidized species and dimer) illustrated in Table 1. The spectra included in the fitting are displayed in different colors: each spectrum is multiplied by a factor proportional to the weight adopted in the fit.

As is shown in Figure 7, fitting of an experimental IR spectrum of modified TMP was attempted by combining the theoretical spectra of five model compounds: TMP, DMP-CHO, DMP-COOH, DMP-COOH H-bonded dimer, and (DMP)_2_. The resulting fitting curve (red) reasonably describes the major experimental features with the exception of few spectral regions. In particular, a broad absorption feature, at low frequency, of the strong C=O stretching features is not predicted by theory. In addition, in the region between 1200 and 900 cm^−1^, some theoretically predicted bands do not find an experimental counterpart. There are several reasons which justify such findings. The experimental spectrum is fitted with the weighted sum of the spectra of few model molecules in their optimized lower energy geometry and the contribution of conformational isomers—which in principle could be present—is not considered. Moreover, theoretical spectra describe isolated molecules (or dimers) *in vacuo* and intermolecular interactions occurring in the solid phase are not described at all.

However, it is possible to say that the direct comparison of the experimental spectrum with theoretical spectra of the individual components corroborates the assignment of several marker bands, proposed on the basis of the discussion above. Interestingly, the fitting seems to suggest that MP-(CHO)_2_ is most suitable than DMP-CHO for the description of several features associated to TMP oxidation.

TMP sample treated at 130 °C in the presence of catalytic amount of HSAG is a peculiar case. It reveals spectral features, both ^1^H NMR (as mentioned above) and FT-IR, which are absent in spectra of samples treated at all the other temperatures. These results were reproduced in several experiments. It was hypothesized that oxidation led to the formation of amidic species: ((Z)-*N*-(4-oxobut-2-*en*-2-yl)formamide) (Amide-CHO) and ((Z)-3-formamidobut-2-enoic acid) (Amide-COOH) (see Table 2). DFT computed IR spectra of Amide-CHO and Amide-COOH allowed to fit the peaks observed in the experimental spectra, which could not be justified with either TMP or (DMP)_2_ or oxidized species (Section SI-2, Figures SI-6 and SI-7). To account for such species, the pyrrole ring opening has to be hypothesized. A mechanism to support such a hypothesis is described in the Supplementary Information (Figures SI-7 and SI-8).

**Figure 8:**
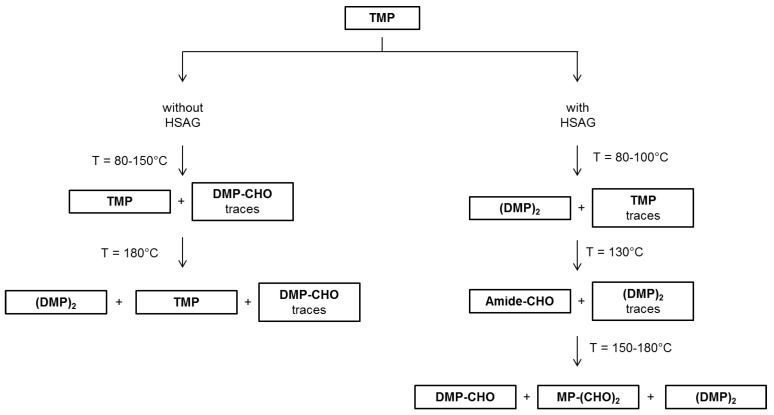
Scheme for the formation of TMP derivatives after thermal treatments at different temperatures, in the absence and in the presence of a catalytic amount of HSAG, from ^1^H NMR analysis of the liquid fraction.

Results discussed so far allow for depicting a picture of what happens when TMP is heated in air, at temperatures from 80 °C to 180 °C. Such a picture is summarized in Figure 8.

Thermal treatments in air of TMP promote TMP oxidation, with formation of aldehydes and dimerization products. TMP derivatives are present in an appreciable amount only when TMP is heated in the presence of catalytic amount of HSAG, whereas are in very low amount (almost traces) when heating is in the absence of HSAG. Temperature increase seems to favor the formation of TMP derivatives. Amidic species were also observed as TMP oxidation products, however only by treating TMP at 130 °C in the presence of HSAG.

TMP oxidation, in the presence of HSAG, can be justified taking into consideration the catalytic effect of this high surface area nanosized graphite [51,56]. Interestingly, in most experiments the oxidation phenomenon seems not to involve the pyrrole ring. It could be hypothesized that oxidation occurs on TMP absorbed on the graphitic substrate and that the π-π interaction protects the aromatic ring from oxidation. Aldehydes and carboxylic acids were found. The dimer formation could be thus justified with the decarboxylation of DMP-COOH. The peculiar result obtained at 130 °C was indeed unexpected: the presence of an amide was detected. This leads to hypothesize the opening of the pyrrole ring, as if the graphitic substrate, in the specific experimental conditions, could not protect the ring from opening and oxidation. However, these are only speculations and further work will be done to explain the experimental result.

### 3.2 Reaction of HSAG with TMP: Solid Fraction.

HSAG/TMP mixtures in equimolar amount were heated in air at 25, 80, 100, 130, 150 and 180 °C respectively. After the thermal treatments, the solid phases were recovered and rinsed with acetone until the washing solvent did not reveal the presence of any organic substance (details are in the experimental part). They were analyzed by means of X-Ray, Raman, FT-IR (performed in transmission in diamond anvil cell (DAC)), XPS and HRTEM analyses.

Results from Raman spectroscopy are provided as Supplementary Information. Raman spectroscopy is largely used for the study of carbonaceous materials [43,46,49,50,51,57,58,59,60,61,62,63]. Attention is particularly focused on two peaks named D and G, located at 1350 cm^−1^ and 1590 cm^−1^ respectively. The G peak is due to bulk crystalline graphite (graphene) and the D peak reveals the presence of either structural defects or confinement (e.g. by edges) of the graphitic layers [60,61,62,63,64]. Spectra of pristine HSAG and HSAG-TMP adduct treated at 180 °C are reported in Figure SI-9. The G and D bands are present in both spectra with similar intensity, thus indicating that HSAG is a disordered graphitic material because of the large amount of edges due to its nano-size. The comparison between the two spectra in Figure SI-9 suggests that the thermal treatment of HSAG with TMP does not appreciably alter the bulk structure of the graphitic system.

**Figure 9:**
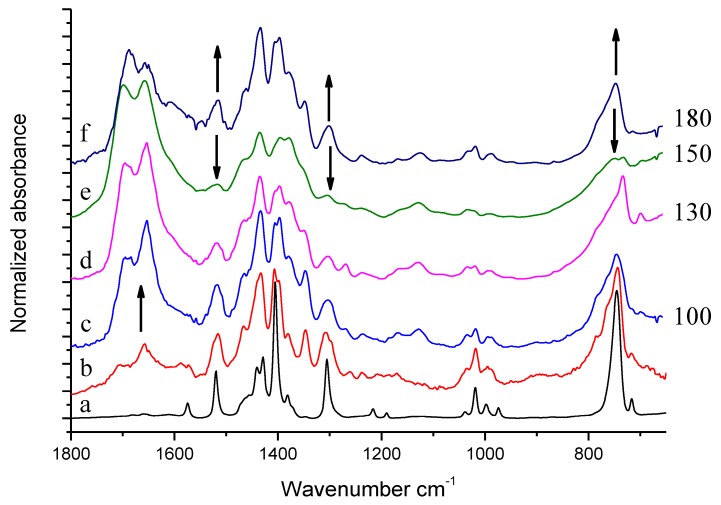
Experimental spectra of pristine TMP (**a**), reference spectrum of modified TMP (sample treated at 130 °C-residue after evaporation)) (**b**), solid powder of HSAG/TMP mixture isolated after the reaction of TMP with the catalytic amount of HSAG (**c**)–(**f**). On the right side of the Figure, treatment temperatures (°C) are indicated.

IR spectra of HSAG/TMP mixtures, isolated after the thermal treatments in the presence of catalytic amount of HSAG, are shown in Figure 9: spectra (c), (d), (e), and (f) refer to samples heated at 100, 130, 150 and 180 °C respectively. The spectrum of pristine TMP (a) and the reference spectrum of modified TMP (sample kept at 130 °C and recorded after TMP evaporation) (b) are also shown for comparison.

Spectra (c)–(f) show features which can be mainly ascribed to oxidized species and to (DMP)_2_. This assignment is made on the basis of what discussed above in the text. Remarkably higher absorption intensities can be seen in the C=O stretching region in spectra (c)–(f), with respect to the reference spectrum (b) obtained from the residue after evaporation of a drop of the liquid phase. The upward arrow at about 1650 cm^−1^ indicates the increase of band absorption intensities, from the liquid phase to the solid powder. It seems thus that TMP oxidation products were preferentially adsorbed on HSAG. It is indeed worth noting that samples heated at 100 °C, 130 °C and 180 °C (c), (d), and (f), respectively, reveal very similar spectral features, whereas substantially different appears the spectrum of the sample treated at 150 °C (e). In the spectrum (e), the downward arrows at 1516 cm^−1^, 1330 cm^−1^ and at about 800 cm^−1^ indicate the peaks whose intensity substantially decreases with respect to spectra (c), (d) and (f). Indeed, the band assigned to C=C stretching of the pyrrole ring (at 1516 cm^−1^) and the band assigned to a breathing vibration of the pyrrole ring (at 1301 cm^−1^) remarkably weaken in spectrum (e) and the shape of the structured absorption band in the region 1500–1330 cm^−1^ is modified. Moreover, the CH out of plane bending features below 800 cm^−1^ become very broad and weak. What is indeed intriguing is that the HSAG-TMP sample, passing from 130 °C to 150 °C and then to 180 °C, appears first to change and then to recover its IR spectral features, and thus reasonably recovers its structure.

**Figure 10:**
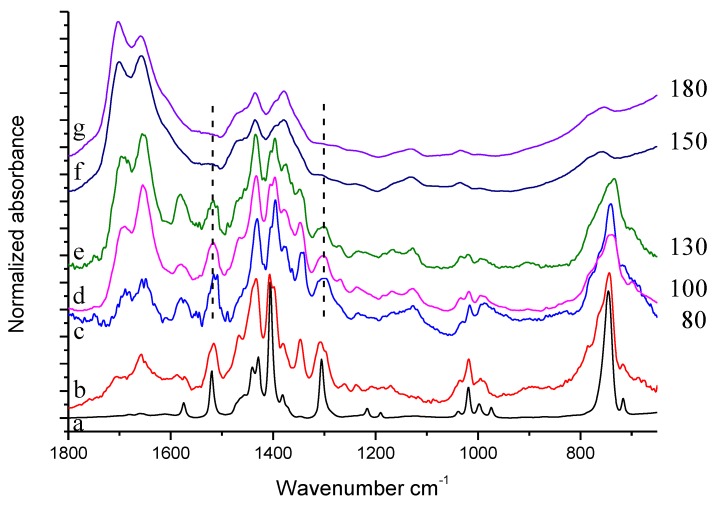
Experimental spectra of: (**a**) TMP, (**b**) reference spectrum of TMP sample treated in air at 130 °C (residue after evaporation), (**c**)–(**g**) solid powder of HSAG/TMP mixture in equimolar amount treated at different temperature.

IR spectra of equimolar mixtures of HSAG and TMP, heated at 80 °C, 100 °C, 130 °C, 150 °C and 180 °C respectively, are shown in Figure 10.

Thermal treatment of HSAG-TMP equimolar mixture leads to modification of spectral features, which are very similar in spectra (c–e), namely in the spectra of the samples treated from 80 °C to 130 °C. For these samples, thermal treatment mainly results in the formation of oxidized species. Spectrum (f) refers to the HSAG/TMP 1:1 sample heated at 150 °C. This spectrum is very similar to the spectrum of HSAG/TMP mixture heated at the same temperature in the presence of the catalytic amount of HSAG, namely to spectrum (e) in Figure 9. Spectrum (g) refers to the HSAG/TMP 1:1 sample heated at 180 °C. By comparing this spectrum (g) with spectrum (f) in Figure 9 (mixture of TMP and HSAG in catalytic amount, heated at the same temperature) a clear difference appears. The equimolar HSAG-TMP sample treated at 180 °C maintains in spectrum (g) the same spectral features of the sample treated at 150 °C, spectrum (f), and does not recover the features of the sample treated at 130 °C, spectrum (e) as in the case of the HSAG/TMP sample, with HSAG in catalytic amount (see spectrum (e) in Figure 9).

**Figure 11:**
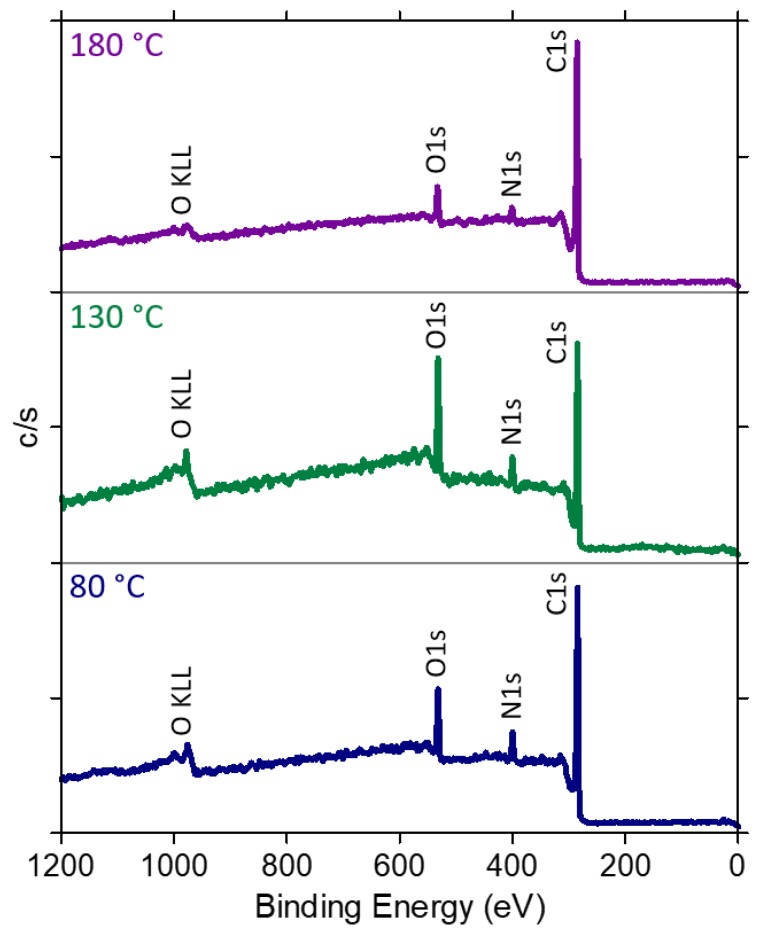
Wide scan XPS spectra of HSAG/TMP equimolar mixtures treated at 80, 130 and 180 °C.

XPS analysis was performed on pristine HSAG and on HSAG-TMP equimolar adducts obtained at 80 °C, 130 °C and 180 °C. It is widely acknowledged that XPS studies on functionalized hybrid materials are very helpful to elucidate the structure of the adducts [65,66,67]. Indeed, XPS analyzes the elemental composition of the samples as well as the types of chemical bonds, providing relevant information. Wide scan XPS spectra are in Figure 11, and C1s core-level spectra are reported in Figure SI-10.

The percentage of C, O, and N atoms for pristine HSAG and HSAG/TMP is reported in Table 3.

The oxygen content in HSAG/TMP samples is larger than in pristine HSAG. Interestingly, the amount of oxygen increases in samples treated at T up to 130 °C and decreases in the sample obtained at 180 °C. The XPS O1s core-level spectra were curve-resolved with two components (Figure SI-11): one at 531.6 eV assigned to C=**O** groups and a second one at 533.1 eV assigned to C–O groups. The C=O/C–O ratio is equal to 0.3, 1.1 and 1.2 for HSAG/TMP mixtures treated at 80 °C, 130 °C and 180 °C, respectively. The presence of oxidized species in HSAG-TMP adducts obtained at T > 80 °C was observed also in FT-IR spectra. XPS findings suggest the occurring, at the highest temperature, of a decarboxylation process.

All the HSAG/TMP samples contain nitrogen, which is not present in pristine HSAG, thus confirming the formation of HSAG/TMP adducts. The ratio N1s/C1s is similar for all HSAG/TMP mixtures, while the ratio N1s/O1s first decreases and then increases with the treatment temperature. The last finding could be as well explained with the occurring of the decarboxylation reaction.

**Figure 12:**
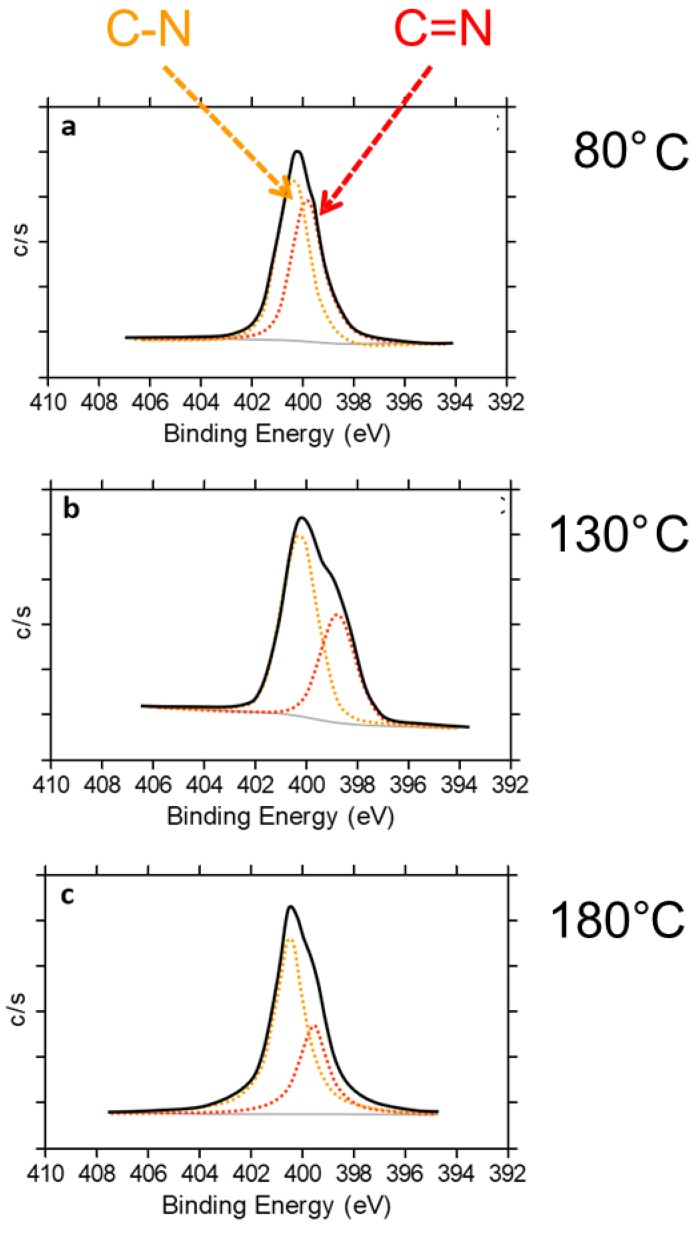
Deconvolution of the N1s peak into two contributions, at 398.9 and 400.3 eV, attributed to C(sp^2^)N (N=C) and C(sp^3^)N (N–C) bonds, respectively, for HSAG/TMP equimolar adducts treated at 80 °C (**a**), 130 °C (**b**), 180 °C (**c**).

Deconvolution of the N1s peak into two contributions, at 398.9 and 400.3 eV (Figure 12), was performed. Such signals were assigned to the photoelectrons emitted by C(sp^2^)N and C(sp^3^)N bonds, respectively. As can be seen in Figure 12, the C(sp^2^)N and C(sp^3^)N ratio (N–C/N=C) increases with the treatment temperature; it is equal to 1.1, 1.7 and 2.0 for HSAG/TMP adducts obtained at 80 °C, 130 °C and 180 °C, respectively.

The structure of HSAG-TMP adducts was also investigated by performing HRTEM analysis on samples isolated from water supernatant suspensions (see the Experimental Part). Figure 12 shows HRTEM micrographs of pristine HSAG (a,c) and HSAG-TMP adduct (b,d) obtained at 180 °C.

**Figure 13:**
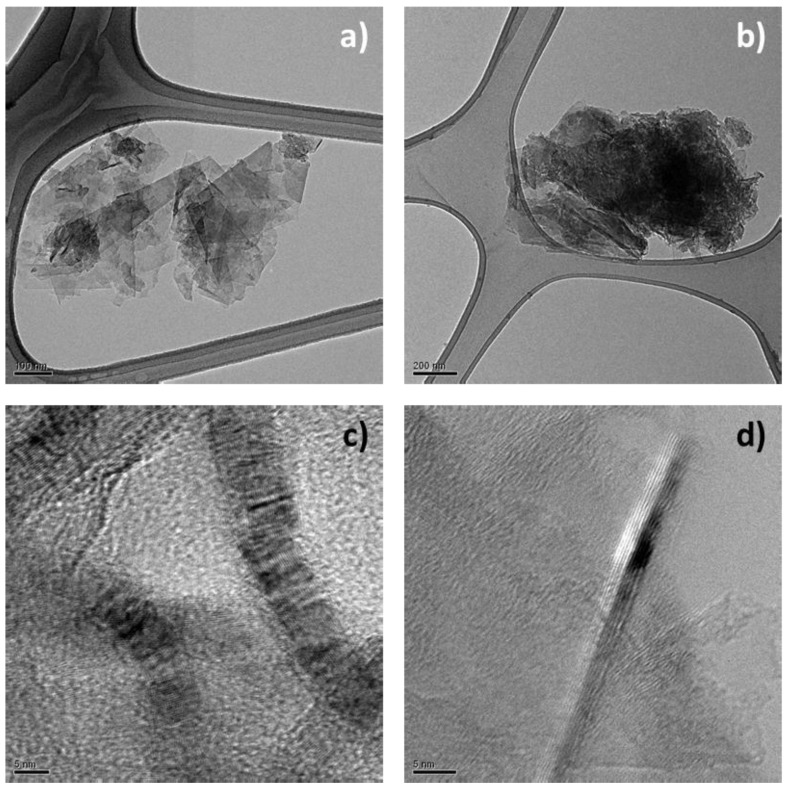
Micrographs of HSAG (**a,c**) and HSAG-TMP adduct obtained at 180 °C (**b,d**). Micrographs are: low magnification bright field TEM (**a, b**), HRTEM images (**c,d**).

Micrographs in Figure 13a,b reveal that the lateral size of pristine HSAG and HSAG-TMP adduct is of the same order of magnitude. Hence, there is no indication in the above pictures that the treatment with TMP leads to appreciable breaking of the graphitic layers. Distribution size of the nanoparticles has not been investigated in the present work. However, functionalization of HSAG has been performed with some different pyrrole compounds and many TEM micrographs have been taken. As already reported, with 2-(2,5-dimethyl-1*H*-pyrrol-1-yl)-1,3-propanediol (SP) as the pyrrole compound [43], the lateral size of the graphene layers was not observed to be modified by the functionalization process.

### 3.3. Mechanism for the Formation of the Adduct Between TMP and Graphitic Substrate

**Figure 14:**
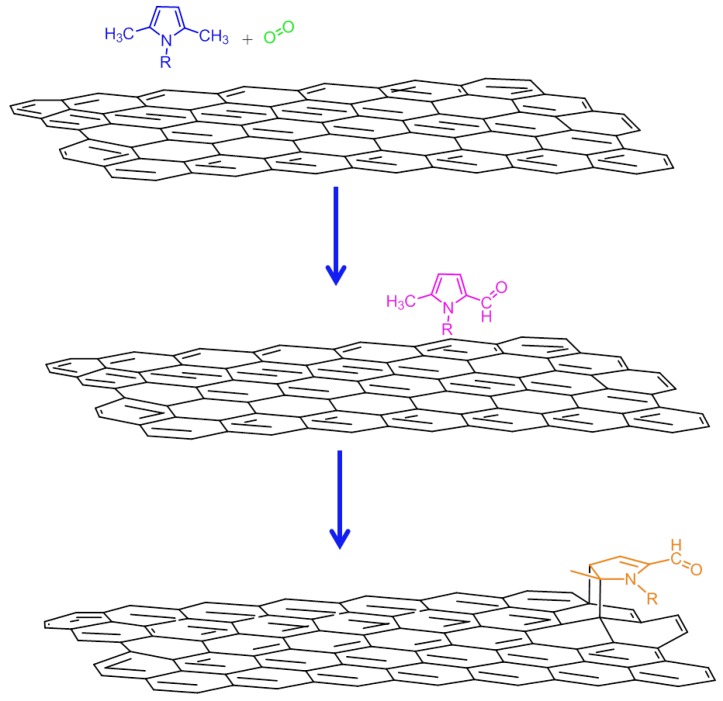
Hypothesized mechanism for the decoration of graphene layers with pyrrole compounds.

The experimental findings and theoretical simulations reported above led us to assume the occurring of a reaction between TMP and the graphitic substrate. Without stretching inferences too far, a reaction mechanism has been elaborated that is able to explain most results: it describes a cascade reaction composed of two steps, a carbocatalyzed oxidation followed by a Diels-Alder reaction as discussed in the following and summarized in Figure 14 and Figure SI-11.

The first step of the reaction is assumed to be the carbocatalyzed oxidation of TMP, which leads to oxidized TMP derivatives. In fact, both FT-IR and XPS findings reveal the presence of oxidized species in HSAG/TMP adducts, heated at T ≥ 80 °C. Oxidized species are also found in ^1^H NMR spectra of liquids extracted from the adducts. In particular, 1,5-dimethyl-1*H*-pyrrole-2-carbaldehyde was isolated and characterized. Hence, it can be reasonably hypothesized that the graphitic substrate promotes the oxidation of TMP, which does not occur in the absence of HSAG, and stabilizes the pyrrole ring, which maintains its aromatic nature.

At T of at least 150 °C, reaction(s) among the oxidized species and the graphitic substrate appear to take place. Such reaction(s) involves the C=C double bonds of the pyrrole ring, as documented by the weakening of their absorption in the IR spectra shown in Figure 9 spectrum (e) and in Figure 10 spectra (f) and (g). A cycloaddition reaction, i.e. a Diels-Alder reaction, can be hypothesized. As is shown in Figure 14, such a reaction should lead to the formation of C–N bonds, in place of C=N bonds. Findings from XPS analysis confirm the occurrence of the cycloaddition reaction.

The Diels-Alder reaction [68] could in principle occur between the double bond of the pyrrole ring, activated by oxidized lateral substituent (either aldehydic or carboxylic acid groups) and the graphitic substrate. The activated pyrrole molecule would act as the dienophile and the graphene layer as the diene.

The reaction between the graphitic substrate and the oxidized pyrrole compound was observed to be reversible, going from 150 °C to 180 °C in the case of TMP heating in the presence of a catalytic amount of HSAG (see spectra (e) and (f) in Figure 9). Such reversibility is compatible with a retro Diels-Alder reaction and can be considered as a retro Diels-Alder. Such a reaction was not observed in the case of the adducts formed using the 1:1 HSAG/TMP molar ratio. Likely, in this case, a higher temperature is required.

The mechanism hypothesized for the reaction of TMP with HSAG involves the Diels-Alder reaction of the graphitic substrate with an oxidized TMP derivative, such as DMP-CHO. The validity of this mechanism was investigated by performing the reaction of TMP with anthracene as a model compound. TMP/anthracene equimolar mixtures were kept (as described in the experimental part, Figures SI-12–Si-17) at the following temperatures 25, 80, 100, 130, 150 and 180 °C. FT-IR spectra were taken on the powders isolated at the end of each thermal treatment. Experimental details, FT-IR and DFT spectra are provided as Supplementary Information (SI-15 and SI-17). In brief, findings were obtained in line with the expected reaction, though in the presence of by-products.

## 4. Conclusions

The decoration of a few layers graphene system was obtained by simply heating a mixture of a high surface area nanosized graphite and a pyrrole compound such as 1,2,5-trimethylpyrrole, in the presence of air and in the absence of solvent or catalyst. This work proposes that a cascade reaction, made by carbocatalyzed oxidation and Diels-Alder cycloaddition, occurs when a pyrrole compound is heated in the presence of a graphitic substrate, in oxidizing conditions.

Such a cascade process during the thermal treatment left substantially unaltered the bulk structure of HSAG, as revealed by Raman spectroscopy, as well as its lateral size, as revealed by HR-TEM analysis, and is able to explain the formation of stable adducts between pyrrole compounds and sp^2^ carbon allotropes, as reported in previous papers [43,46,48].

^1^H NMR, IR spectroscopy in conjunction with DFT calculations, and XPS analyses allowed to identify the species produced by the thermal treatment. HSAG/TMP powders heated at a T between 80 °C and 130 °C revealed the presence of TMP oxidized derivatives based on a pyrrole ring with preserved aromaticity. When heating was done at T ≥ 150 °C, clear indications of the reaction between oxidized TMP and the graphitic substrate have been obtained. IR spectra, supported by DFT calculations, revealed the reaction of intra-annular double bond. Diels-Alder cycloaddition reaction between the dienophile pyrrole compound and the diene substrate appears to occur. This conclusion is supported by the results of XPS. Indeed, the reduction of the C(sp^2^)N/C(sp^3^)N ratio is compatible with the loss of aromaticity of the pyrrole ring and the formation of the final oxidized product shown in Figure 13. The addition of oxidized TMP was observed to be reversible for T > 150 °C, when TMP was heated in the presence of a catalytic amount of HSAG. In the case of the equimolar HSAG-TMP adduct, it appears that the graphitic substrate hinders a retro Diels-Alder reaction.

Functionalization of carbon allotropes can be thus performed with a simple and sustainable process, which does not produce waste. This paves the way for large scale applications.

## Figures and Tables

**Table 1:** Values of the wavenumbers corresponding to the absorption peaks of the most intense IR transitions: comparison between experimental and computed values (B3LYP/6-311++G(d,p)). Vibrational assignments are based on computed eigenvectors analysis.

Experimental Wavenumber (cm^−1^)	From DFT (scaled values, f = 0.978) Wavenumber (cm^−1^)	Vibrational Assignment
1575	1574	anti-symm C=C stretching
1520	1517	symm C=C stretching
1441 1429	1460 1450 1435	CH_3_ bending
1405	1403	CH_3_ umbrella, symm CN stretching
1382	1385	CH_3_ umbrella
1346	1358	anti-symm CN stretching
1305	1299	Ring breathing
1216	1212	CH (sp^2^) in-plane wagging
1190	1184	C–N–C bending, N–CH_3_ stretching
1220 (vw, broad)	1113	CH_3_ rocking
1038	1038	CH_3_ rocking
1019	1019	in-plane CH wagging
998	993	CH_3_ rocking
974	966	CH_3_ rocking + ring torsion
746	741	CH opla
717	706	collective symmetric CN stretching

**Table 2:** Structures of model molecules (and complexes) selected for DFT spectra prediction.

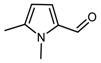 **DMP-CHO** (1,5-dimethyl-1*H*-pyrrole-2-carbaldehyde)	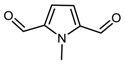 **MP-(CHO)_2_** (1-methyl-1*H*-pyrrole-2,5-dicarbaldehyde)
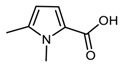 **DMP-COOH** (1,5-dimethyl-1*H*-pyrrole-2-carboxylic acid)	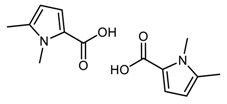 **DMP-COOH** dimer (1,5-dimethyl-1*H*-pyrrole-2-carboxylic acid)
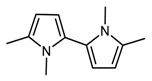 **(DMP)_2_** (1,1′,5,5′-tetramethyl-1*H*,1′*H*-2,2′-bipyrrole)	
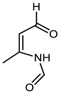 **Amide-CHO** ((Z)-*N*-(4-oxobut-2-en-2-yl)formamide)	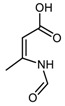 **Amide-COOH** ((Z)-3-formamidobut-2-enoic acid)
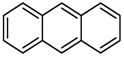 **A** (Anthracene)	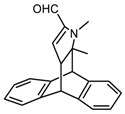 **A/DMP-CHO adduct** (12,13-dimethyl-10,11,12,13-tetrahydro-9*H*-9,10-[2,3]epipyrroloanthracene-14-carbaldehyde)

**Table 3:** Atomic percent concentration and concentration ratios deducted from XPS spectra for pristine HSAG and HSAG/TMP equimolar mixtures treated at 80 °C, 130 °C and 180 °C.

Sample	C1s (at. %)	O1s (at. %)	N1s (at. %)	O1s/C1s	N1s/O1s	N1s/C1s
HSAG	95.8	4.2	0	0.04	0	0
HSAG/TMP 80 °C	81.8	10.8	7.4	0.13	0.68	0.09
HSAG/TMP 130 °C	76.6	15.9	7.5	0.21	0.47	0.1
HSAG/TMP 180 °C	87.9	6.5	5.6	0.07	0.86	0.06

## Supplementary Materials

The following are available online at http://www.mdpi.com/2079-4991/9/1/44/s1.
