# Supplementary material for: Domino Reaction for the Sustainable Functionalization of Few-Layer Graphene
Source: Nanomaterials (Basel). 2018 Dec 30;9(1):44. doi: 10.3390/nano9010044 (PMC6359401; doi:10.3390/nano9010044)
Supplement: Supplementary file 1 [file nanomaterials-09-00044-s001.pdf]

nanomaterials

Supplementary Information

Domino Reaction for the Sustainable Functionalization of Few-Layer Graphene

Vincenzina Barbera [1,*], Luigi Brambilla [1,*] , Alberto Milani [1], Alberto Palazzolo [1], Chiara Castiglioni [1], Alessandra Vitale [2], Roberta Bongiovanni [2] and Maurizio Galimberti [1,*]

1 Politecnico di Milano, Department of Chemistry, Materials and Chemical Engineering "G. Natta", piazza Leonardo da Vinci, 32-via Mancinelli 7, 20131 Milano, Italy; alberto.milani@polimi.it (A.M.), alberto.palazzolo@cea.fr (A.P.), chiara.castiglioni@polimi.it (C.C.)
2 Politecnico di Torino, Department of Applied Science and Technology, Corso Duca degli Abruzzi 24, 10129 Torino, Italy; alessandra.vitale@polito.it (A.V.); roberta.bongiovanni@polito.it (R.B.)
* Correspondences: vincenzina.barbera@polimi.it (V.B.); luigi.brambilla@polimi.it (L.B.); maurizio.galimberti@polimi.it (M.G.); Tel.: +39-02-23994746 (V.B. & M.G.); Tel.: +39-02-23994718 (L.B.)

SI-1—Thermogravimetric analysis: TGA tests under flowing N_2 (60 mL min^{-1}) were performed with a Mettler TGA SDTA/851 instrument according to the standard method ISO9924-1.

Sample (10 mg) was heated from 30 to 300 °C at 10°C min^{-1}, kept at 300 °C for 10 min, and then heated up to 550 °C at 20 °C min^{-1}. After being maintained at 550 °C for 15 min, it was further heated up to 650 °C and kept at 600 °C for 10 min under flowing air (60 mL min^{-1}).

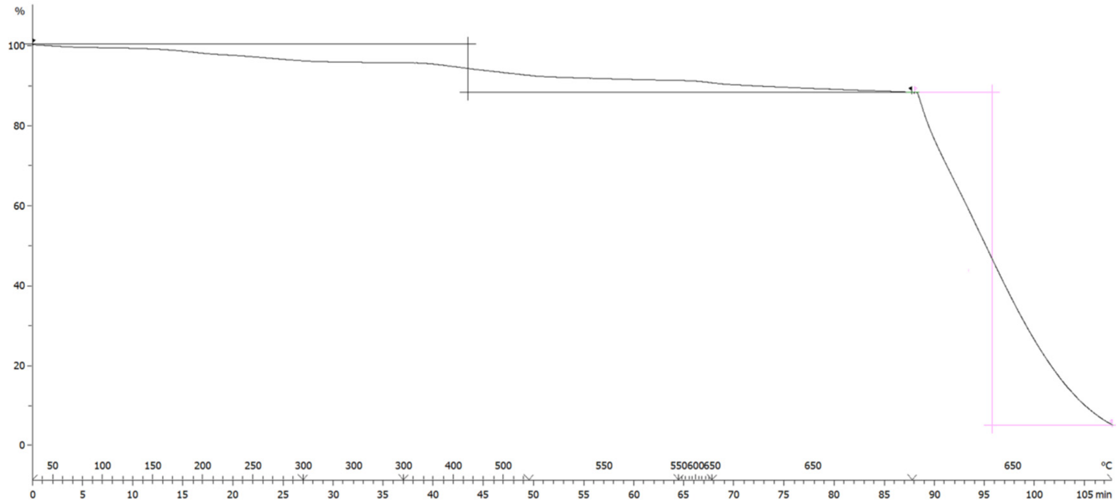

Figure SI-1. TGA thermograph of HSAG functionalized with TMP at 180°C.

TGA revealed mass loss for HSAG functionalized with TMP at 180 °C (HSAG/TMP), below 700 °C, as it is shown by trace in Figure. SI-1. For HSAG/TMP sample, a three-step decomposition profile can be observed. The first and second mass losses below 150 °C and between 150 °C and 700 °C could be attributed to the decomposition of oxygen and nitrogen groups and removal of alkenyl groups. Combustion with oxygen of graphite occurs for temperatures higher than 600 °C.

Experimental Part

About 0.1 µL of the liquid part of TMP/HSAG sample was dissolved in 1 mL of CH_2Cl_2. The resulting solution was injected into a 6890A Series GC System, G1530A model, from Agilent Technologies gas chromatograph. The column was J&W GC Column HP-5MS, 19091S-433 model, from Agilent Technologies, with 5%-Phenyl)-methylpolysiloxane as stationary phase, with 30 m as

the length, 0.25 mm as the internal diameter and 0.25 µm as film thickness. Helium (He) was the carrier gas at a constant flow rate of 1.2 mL/min. The GC was interfaced with 5973N Mass Selective Detector, G2577A Model, mass detector, from Agilent Technologies, operated under the EI mode (70 eV) using an autotune file.

Experimental conditions for the oven were as follows. Initial temp: 40 °C, Initial time: 1.00 min, Equilibration time: 0.50 min. Ramps: 2 °C/min up to 45 °C, 1 min isotherm, 10 °C/min up to 200 °C, 1 min isotherm, 20 °C/min up to 280 °C, 10 min final isotherm.

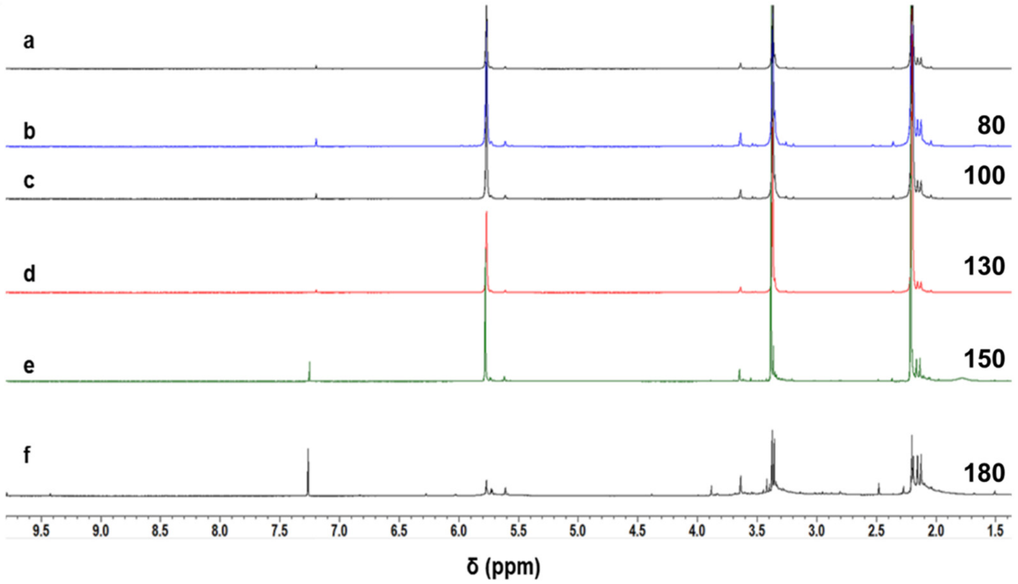

Figure SI-2. ^{1}H NMR spectra (400 MHz, $CDCl_3$) of pristine TMP (a) and the liquid product obtained by heating TMP in the absence of catalytic amount of HSAG (b–f). On the right side of the Figure, treatment temperatures (°C) are indicated.

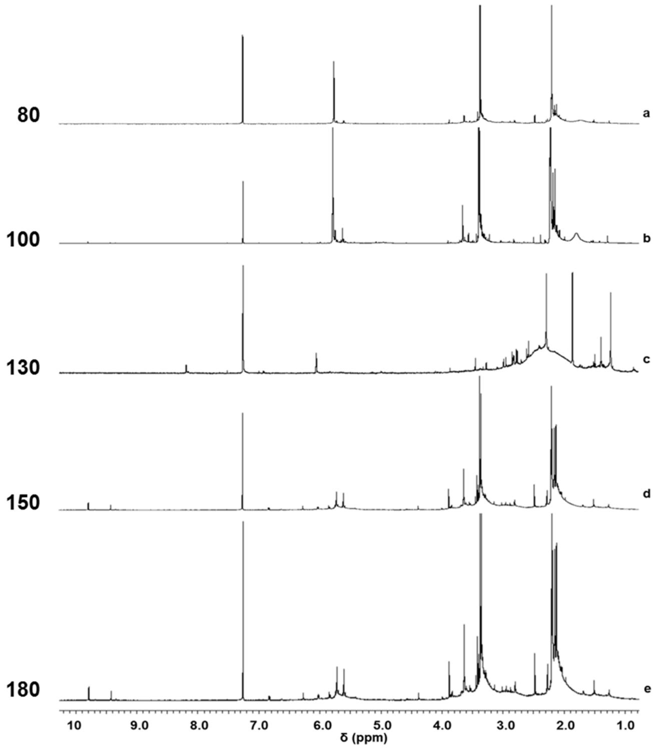

Figure SI-3. ^{1}H NMR spectra (400 MHz, $CDCl_3$) of the liquid product obtained by heating TMP in the presence of catalytic amount of HSAG (a–e). On the left side of the Figure, treatment temperatures (°C) are indicated.

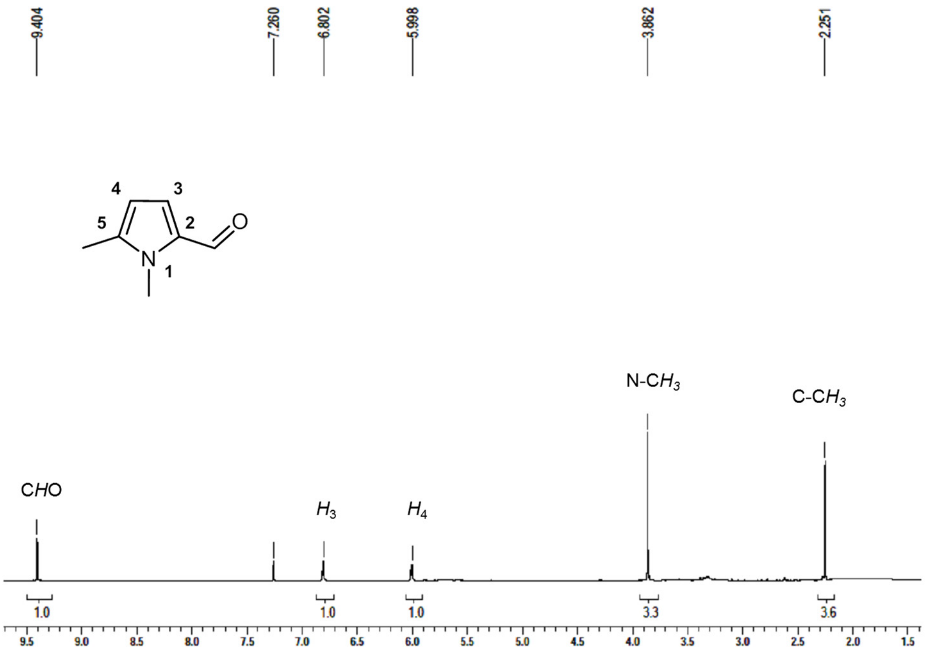

Figure SI-4 ^{1}H NMR spectrum (400 MHz, $CDCl_3$) of DMP-CHO isolated from the liquid product obtained by heating TMP in the presence of catalytic amount of HSAG at 150 °C. (1,5-dimethyl-1*H*-pyrrole-2-carbaldehyde)

In Figure SI-5 are reported the GC/MS chromatograms of TMP samples threated in the presence of 1% w of HSAG at 25 °C (a), 100°C (b) and 150 °C (c).

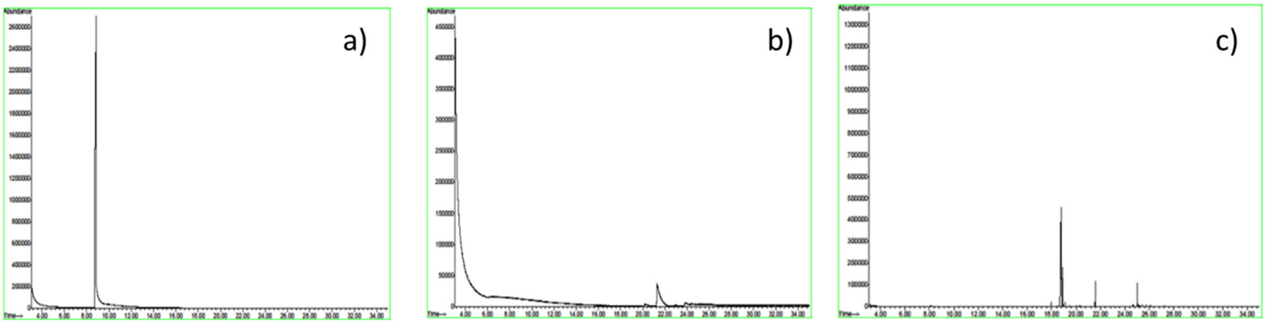

Figure SI-5. GC/MS chromatograms of TMP samples threated in the presence of 1% w of HSAG at 25 °C (a), 100 °C (b) and 150 °C (c).

The sample treated at 25 °C revealed in the chromatogram (a) a peak whose mass was compatible with DMP (9.10 min as retention time), whereas the sample treated at 100 °C showed a peak (Figure SI-5b) whose mass was compatible with DMP_2 (21.8 min as retention time). The sample treated at 150 °C gave rise to a chromatogram with a mixture of products: their mass were those of the following products: DMP-CHO, DMP_2 and $MP(CHO)_2$, with retention times of 18.9, 21.8 and 24.5 min, respectively.

These findings are in agreement with what revealed by ^{1}H-NMR analysis and IR analysis supported by DFT calculations.

SI-2—Formation of amidic species: ^{1}H NMR data and reaction schemes.

In Figure SI-6 it is reported the experimental ^{1}H NMR spectrum of the liquid part from the mixture reaction of TMP after treatment at 130 °C in the presence of a catalytic amount of HSAG.

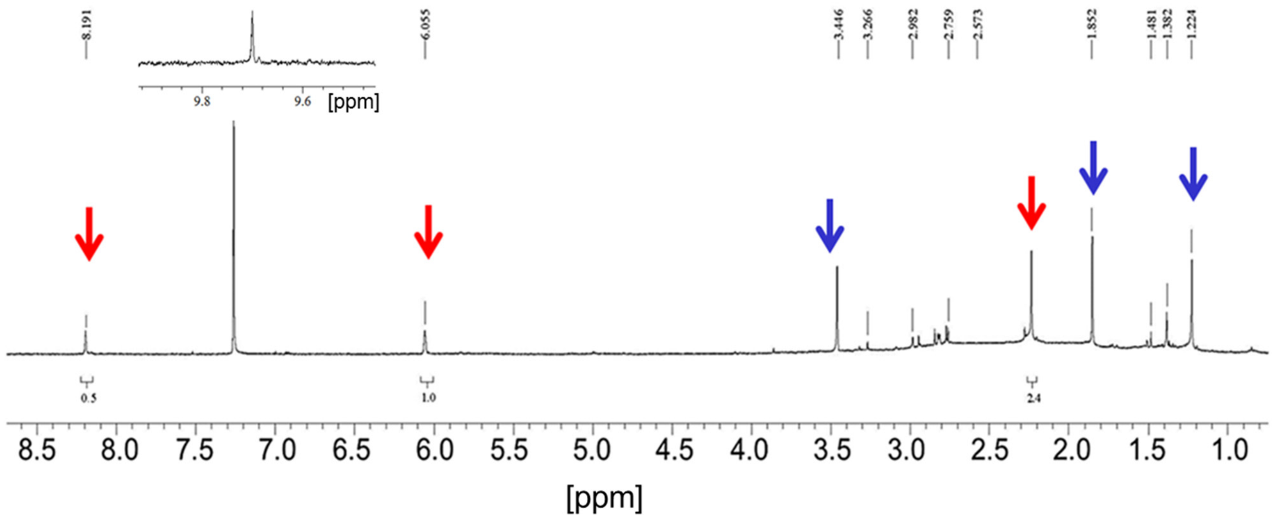

Figure SI-6. ^{1}H NMR spectrum (400 MHz, $CDCl_3$) of the liquid sample after treatment of TMP after treatment at 130°C in the presence of a catalytic amount of HSAG. The arrows indicate peaks due to protons from amide-CHO (red) and oligopyrrole species (blue).

The ^{1}H NMR spectrum exhibits three main resonances (red arrows), at δ = 8.19, 6.05 and 2.26 ppm, which were assigned to the amidic proton, alkenyl C–H proton and the methyl group respectively. However, an examination of the low field region of the spectrum reveals the presence of other methyl proton resonances as singlets at 3.44, 1.85 and 1.20 ppm (blue arrows). These features could be ascribed to the presence of the oligopyrroles .

The formation of the amidic compounds can be explained by taking in account a complex mechanism which involve:

a) the formation of 5-methyl-1*H*-pyrrole-1,2-dicarboxylic acid (MP-$(COOH)_2$)

b) the decarboxylation of MP-$(COOH)_2$ to MP

c) the singlet oxygen addition reaction to MP.

To account for the experimental results discussed so far, mechanism for the formation of amidic species is proposed as follows. Reaction paths for the subsequent benzylic oxidation, decarboxylation and ring opening reaction of TMP are reported in Figure SI-7.

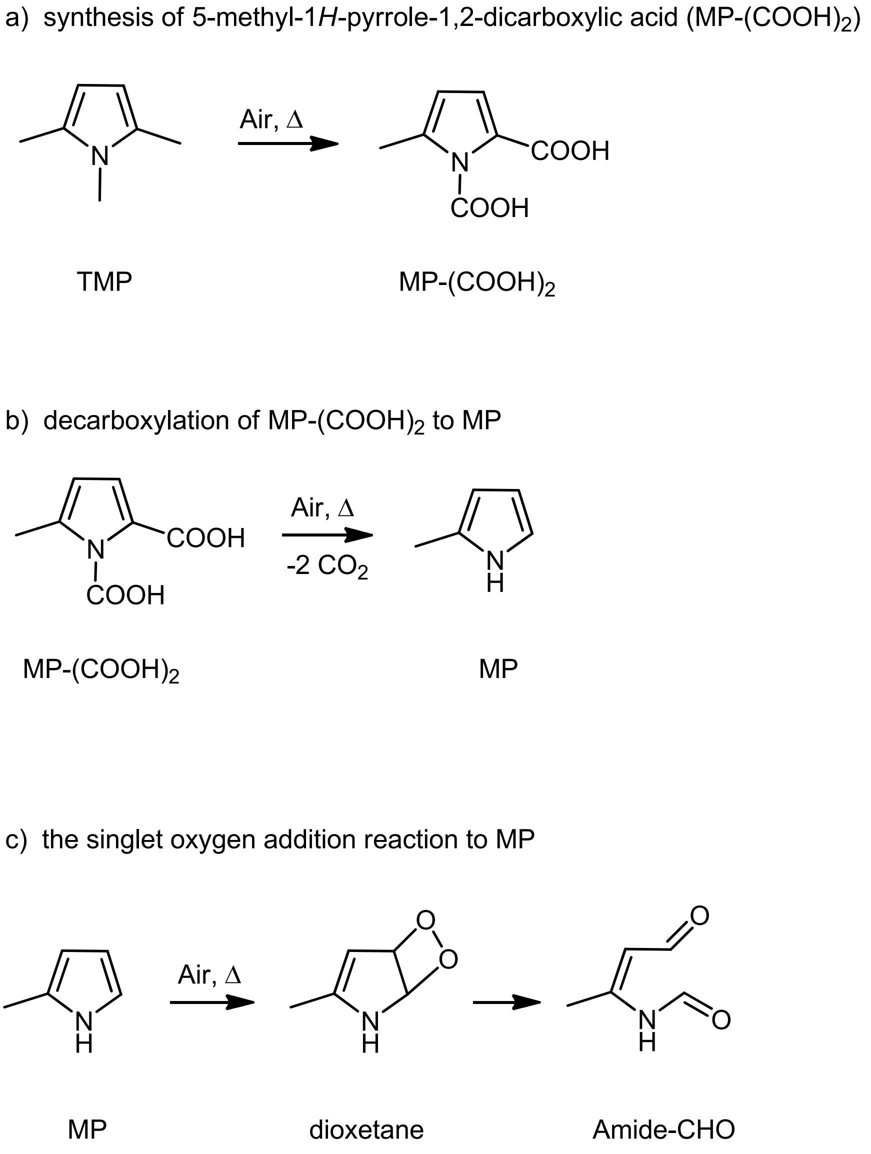

Figure SI-7. Proposed synthetic pathway for the ring opening reaction of TMP.

Singlet oxygen, which is normally generated from molecular oxygen (air) in the presence of visible light, is capable of oxidizing a wide range of substrates. Photooxidations of pyrroles often give a mixture of products derived from both [1,2]- and [1,4]-oxygen additions. This reaction is usually performed in the presence of a photosensitizer molecule, such as methylene blue, and by using a 400 watt tungsten lamp as the light source.

In our reaction conditions, the pyrrole ring and the oxygen molecules are both adsorbed on the graphitic material. In this case, the pyrrole ring results involved through a weak interaction with the graphene layer, with a possible effect of protection of the pyrrole ring. Indeed, this intimate interaction makes the ring less reactive: the first oxidation process takes place on the sp^3 carbon located on the alfa position (benzyl-like).

This behavior is not unusual on benzyl-like molecules: carbon materials are able to transform allylic/benzylic hydrocarbons into corresponding hydroperoxides, ketones, alcohols, carboxylic acids, or their derivatives.

TMP undergoes first a double oxidation of CH_3 in alfa position and on the nitrogen (Figure SI-7a) and after a decarboxylation (Figure SI-7b). The so obtained monomethyl derivative (MP) reacts with the singlet oxygen directly on the ring moiety: The [1,2]-addition occurs between the dioxygen and the less substituted carbon-carbon double bond in alfa position (MP). The so obtained

dioxetane intermediate undergoes a decomposition process, which allows at obtaining the ring-opened compound Amide-CHO (Figure SI-7c).

It is known that aldehydes are easily oxidized to carboxylic acid: in particular, Amide-CHO could undergo a further oxidative process in the presence of oxygen. This kind of carboxylic acid could decarboxylate spontaneously and in fact, signals at 11 ppm are not well detectable in the NMR spectrum (Figure SI-6).

SI-3- Mechanism for the formation of the adduct between TMP and graphitic substrate. Starting from these studies we hypothesize a plausible oxidation mechanism of the pyrrole ring (Figure SI-8).

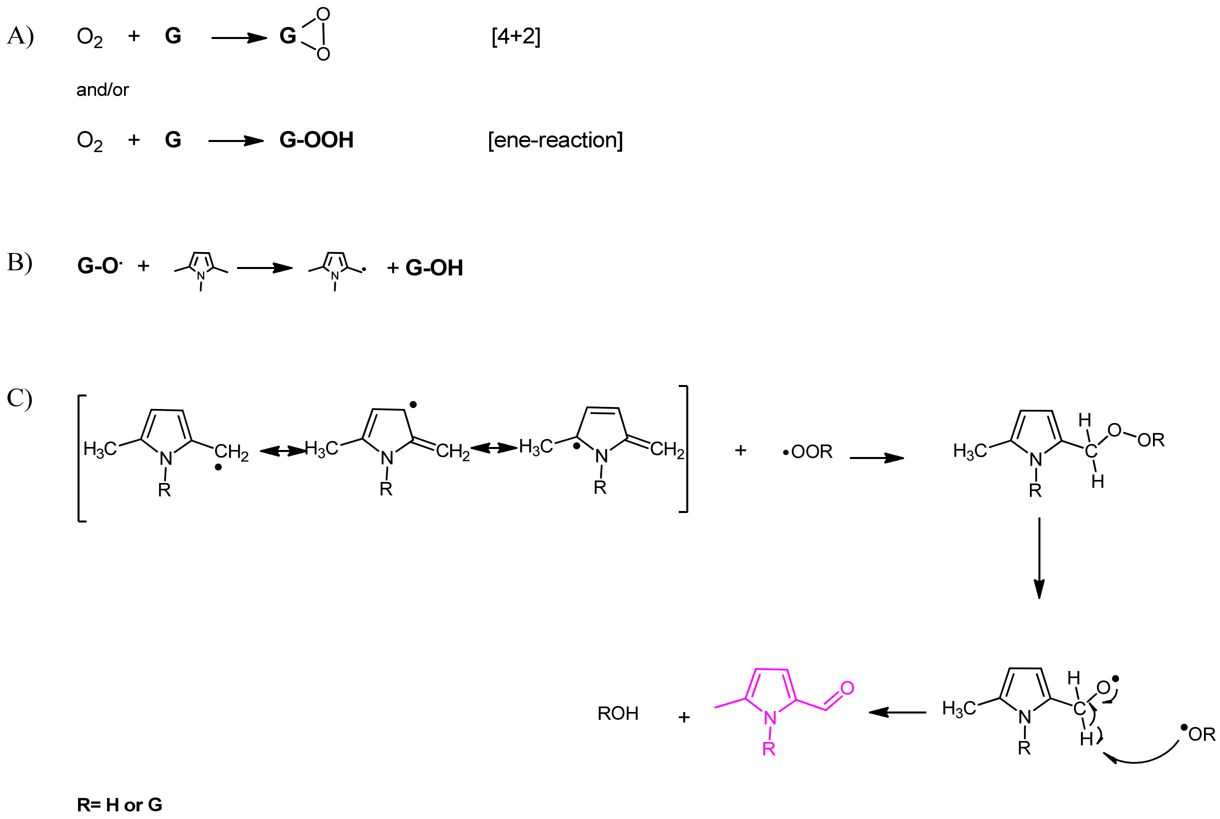

Figure SI-8. Possible mechanism for the carbocatalyzed oxidation of TMP.

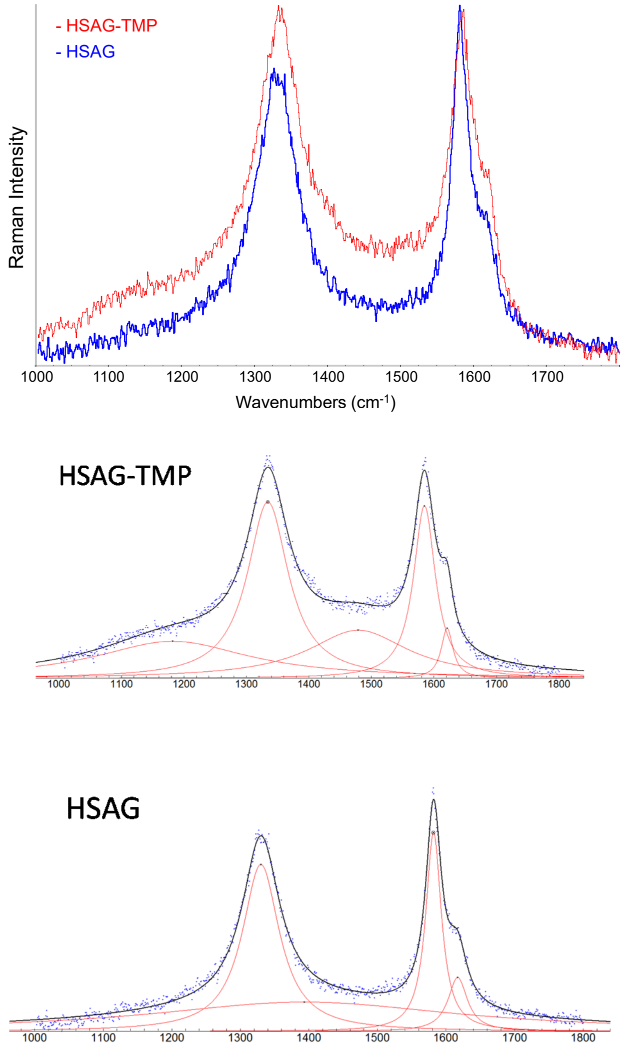

Figure SI-9. Raman spectra of HSAG (blue) and HSAG-TMP (red) excited at 632.8 nm and their curve fitting deconvolution. The intensity ratio I_D/I_G obtained by the fitting (see table) is practically the same for the two samples, thus confirming that the thermal treatment of HSAG with TMP does not appreciably alter the bulk structure of the graphene layers. The remarkable increase in the FWHM observed for the G peak in HSAG-TMP, is not fully rationalized. It can be tentatively ascribed to some disorder in the layers stacking-and possibly to a decrease of the number of layers in the stack-induced by the treatment.

Table SI-1. Raman spectra deconvolution values of HSAG and HSAG-TMP excited at 632.8 nm.

	PeakType	Center	Height	Area	FWHM	D/G
**HSAG-TMP**	**Lorentzian**	**1584.59**	**0.774976**	**51.9917**	**42.7097**	
	**Lorentzian**	**1334.06**	**0.792552**	**99.5652**	**79.976**	1.9
	Lorentzian	1620.85	0.224795	8.11954	22.9945	
	Lorentzian	1478.65	0.216207	66.3017	195.225	
	Lorentzian	1181.67	0.166287	78.7077	301.327	
**HSAG**	**Lorentzian**	**1581.58**	**0.81623**	**35.6193**	**27.7813**	
	**Lorentzian**	**1329.73**	**0.685137**	**71.2746**	**66.2273**	2.0
	Lorentzian	1616.83	0.221934	11.9061	34.1526	
	Lorentzian	1393.25	0.120654	104.843	553.197	

Moreover, according to the results of the deconvolution, the frequency position of the G and D lines result slightly displaced toward higher wavenumbers in the case of HSAG-TMP sample. Due to the very small shifts observed, it is hard to assess the reliability of such information. However, it could be ascribed to modification of the HSAG electronic structure due to the link with TMP.

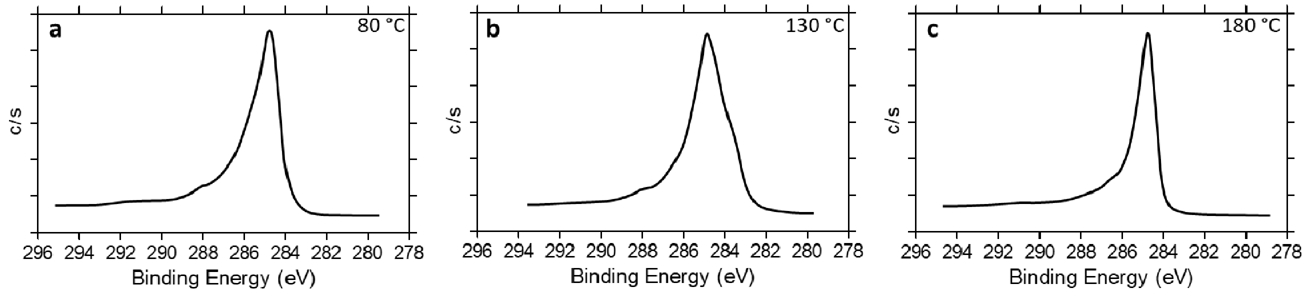

Figure SI-10. XPS C1s core-level spectra of HSAG/TMP equimolar adducts treated at 80 °C (**a**), 130 °C (**b**), 180 °C (**c**).

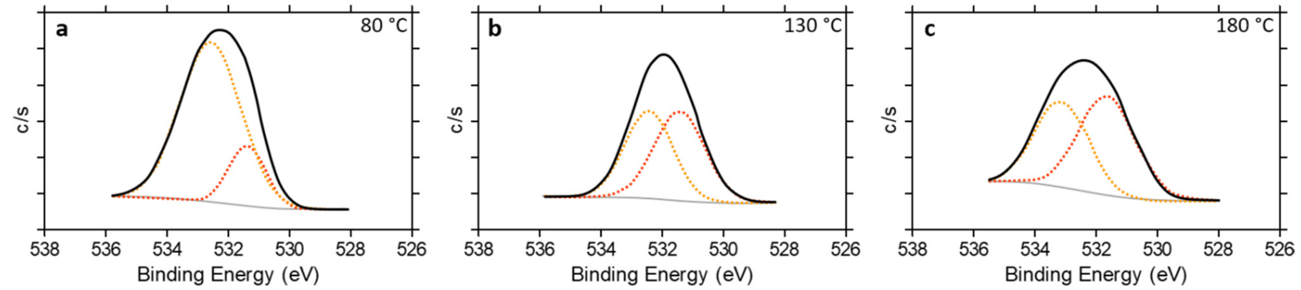

Figure SI-11. Deconvolution of the XPS O1s peak of HSAG/TMP equimolar adducts treated at 80 °C (a), 130 °C (b), 180 °C (c): the component at 531.6 eV is assigned to C=**O** groups and the component at 533.1 eV is assigned to C–**O** groups.

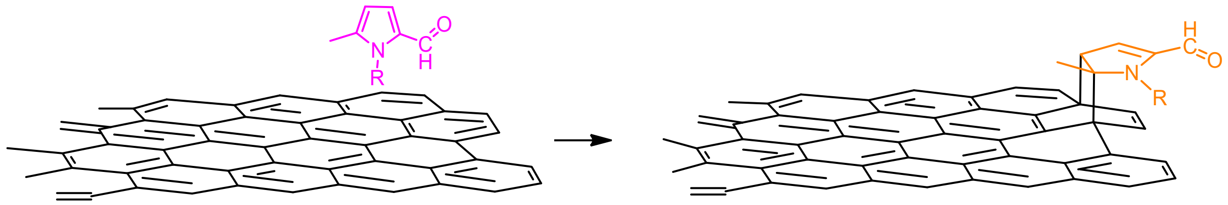

Figure SI-12. Diels-Alder reaction between DMP-CHO and a graphene layer.

SI-4—TMP/Anthracene reaction

Spectra of TMP/anthracene samples heated at temperatures from 80 °C to 130 °C, spectra (b), (c), and (d) in Figure SI-13, show the same features of oxidation species observed in spectra of TMP/HSAG mixtures which were heated at the same temperatures: spectra (c) and (d) in Figure 14 and spectra (c), (d) and (e) in Figure 15.

The spectrum of TMP/anthracene sample heated at T = 150 °C, shown in Figure SI-14, is very similar to spectra of TMP/HSAG mixtures heated at T = 150 °C: spectrum (e) in Figure 14 and spectra (f) and (g) in Figure 15. It is important to note that several sharp lines appearing in the spectrum (b) of Figure SI-14 are due to the presence of unreacted anthracene molecules (spectrum (a)).

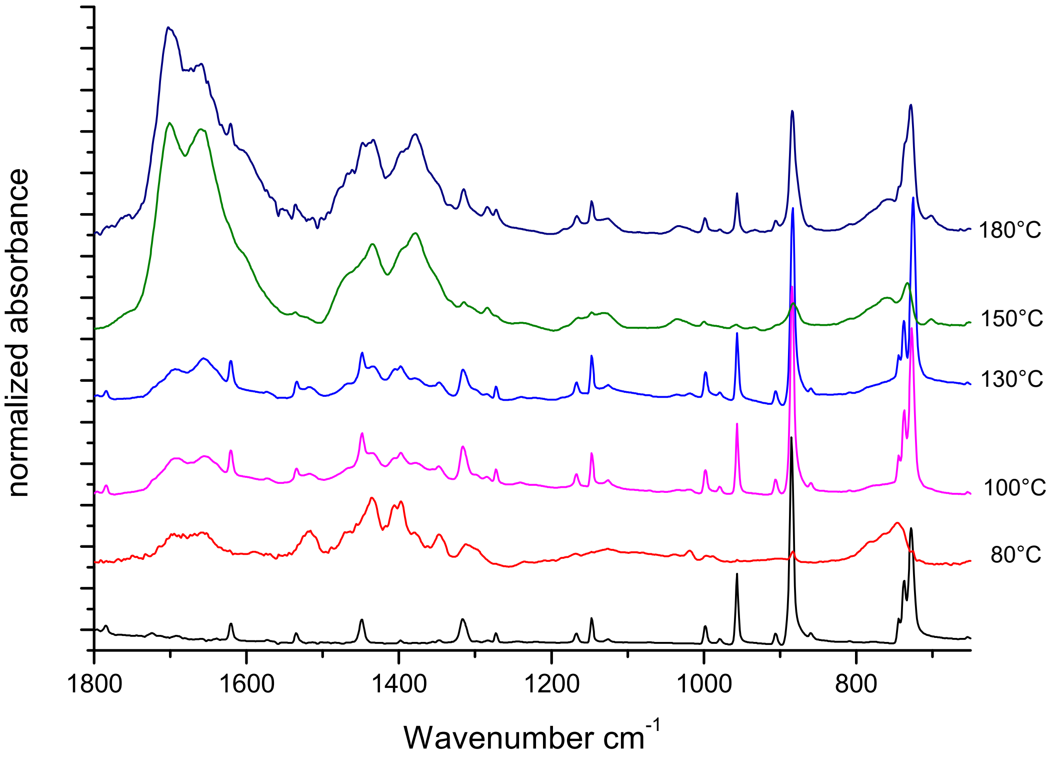

Figure SI-13. Spectra of TMP/anthracene samples treated at temperatures from 80 °C to 180 °C.

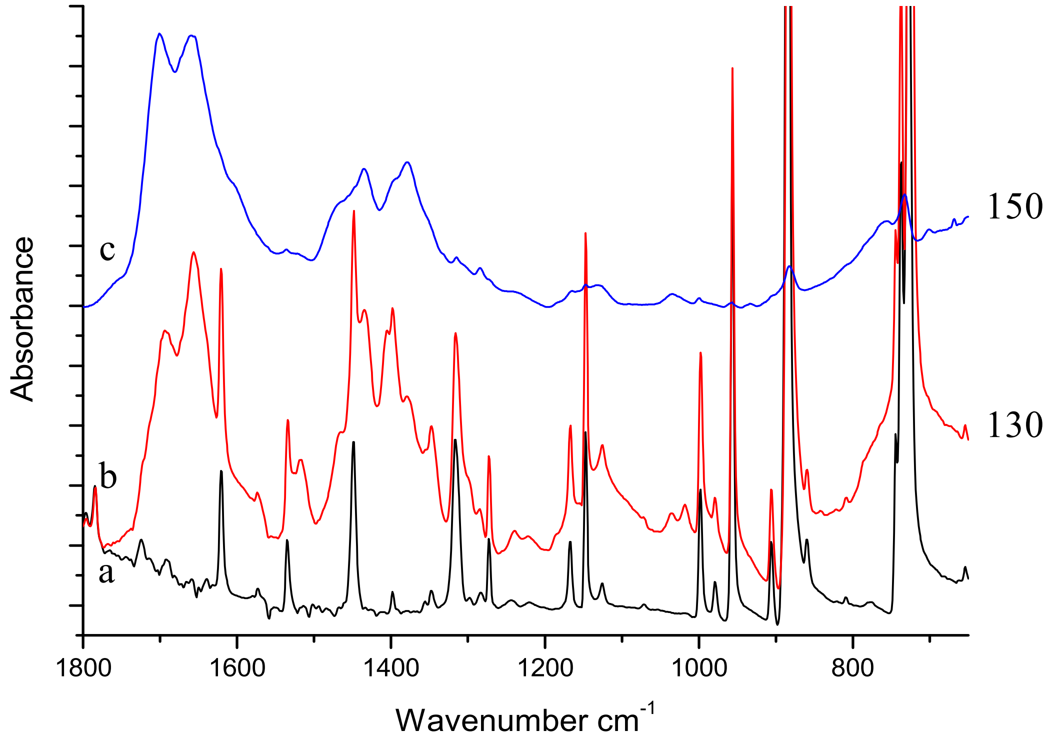

Figure SI-14. Experimental spectra of solid anthracene (a) and of anthracene/TMP mixture treated at 130 °C (b) and at 150 °C (c). On the right side of the Figure, treatment temperatures (°C) are indicated.

It can be thus assumed that TMP, at T = 150 °C, experiences the same modifications in the presence of HSAG and of anthracene. Diels-Alder reaction was hypothesized to occur between anthracene and the oxidized TMP, as it is shown in Figure SI-15.

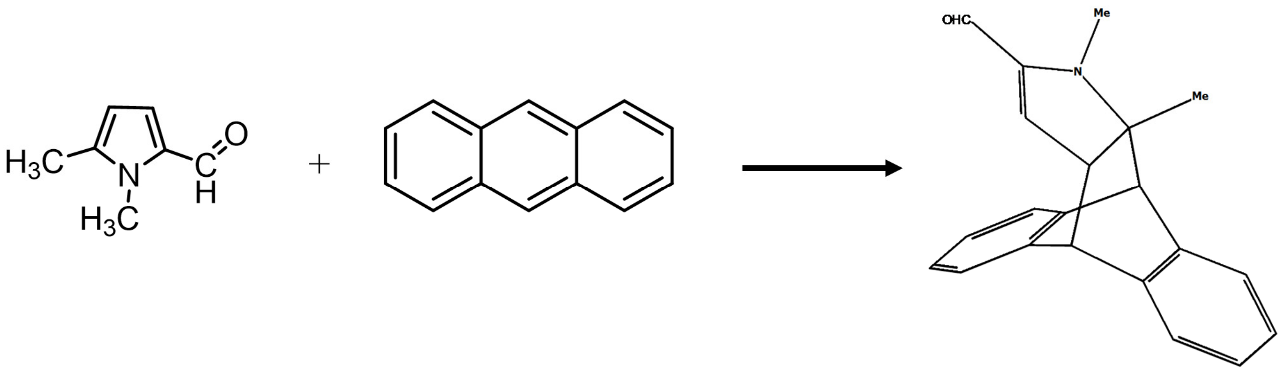

Figure SI-15. Diels-Alder adduct between oxidized TMP and anthracene.

DFT spectra were generated for anthracene and for the covalently bonded adduct of anthracene and DMP-CHO. Theoretical and experimental spectra show very good agreement, as it can be appreciated in Figure SI-1. Spectral features which can be considered as the fingerprint for the adduct formation are focussed in Figure SI-17, where are shown the DFT spectra of DMP-CHO and anthracene-TMP adduct and the experimental spectra of oxidized TMP and the anthracene/TMP mixture treated at 150°C.

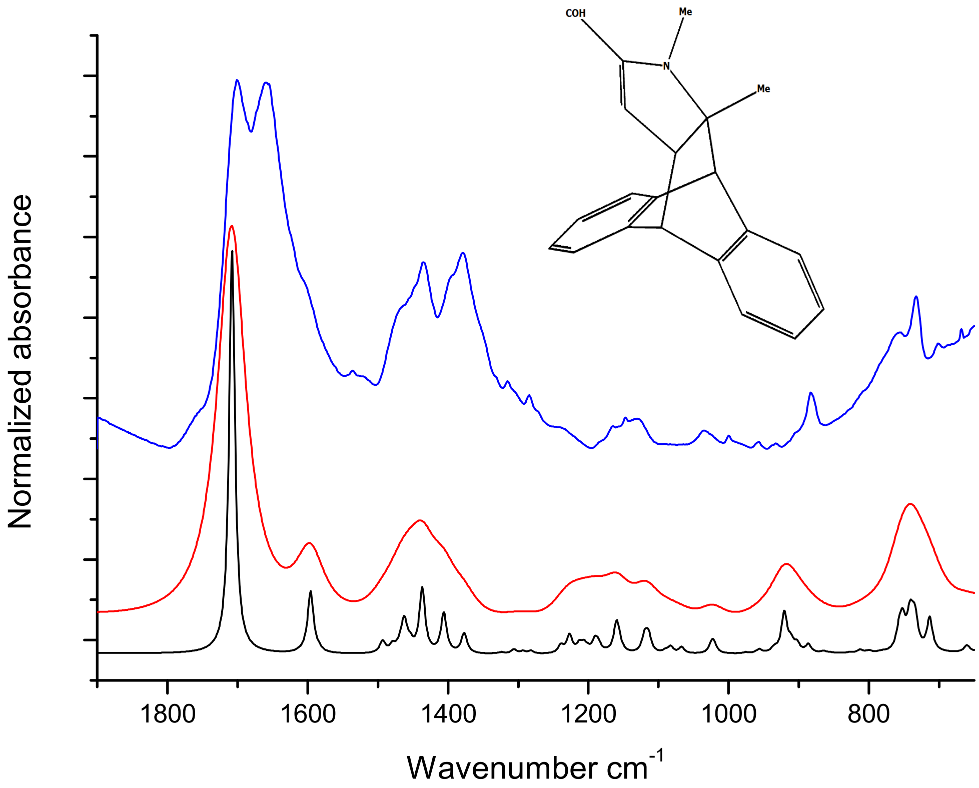

Figure SI-16. Comparison of the experimental IR spectrum of a sample obtained after treatment of Anthracene-TMP mixture at 150 °C (blue line) with the computed spectrum of a model of A/DMP-CHO adduct (black and red lines). The spectrum in red has obtained adopting a wider bandwidth (Γ = 40 cm^{-1}) with respect to the black spectrum (Γ = 10 cm^{-1}) in order to better show the good correspondence between theoretically predicted and experimental pattern. .

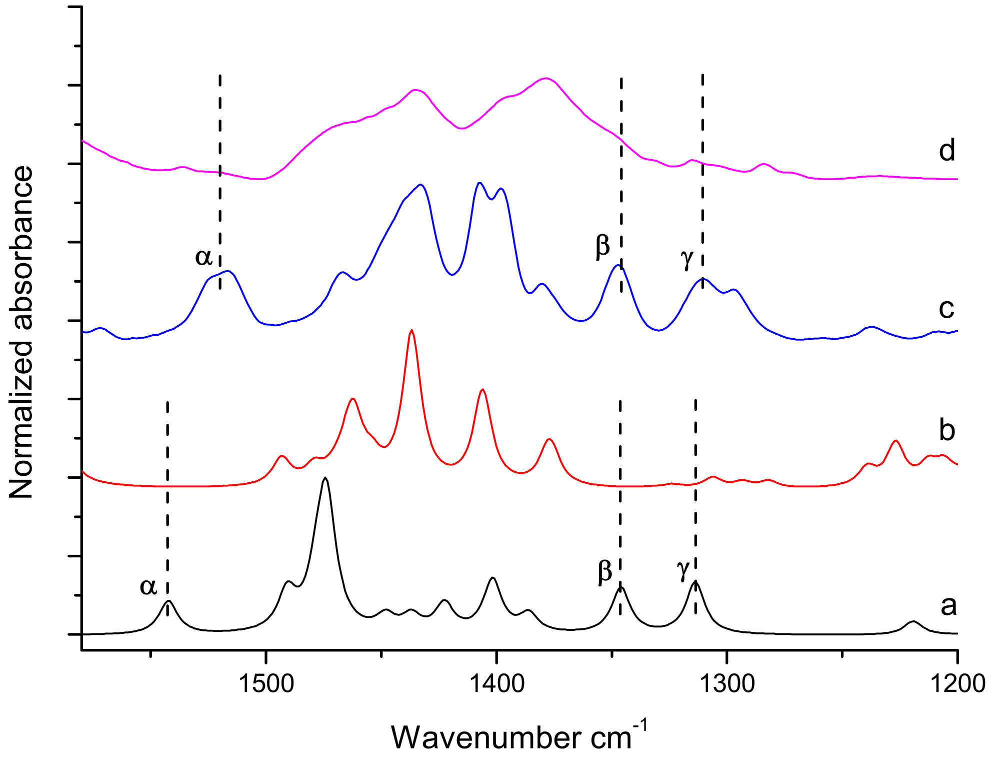

Figure SI-17. Black (a) and red (b) plot at the bottom: comparison between DFT computed IR spectra of DMP-CHO and Anthracene/DMP-CHO adduct. The experimental spectra of Anthracene-TMP mixture at 150 °C (d) and of a sample of modified TMP (*reference* spectrum) (c) are also reported (purple and blue plots at the top).

The computed spectrum of DMP-CHO (a) shows some characteristic features which can be found in the experimental spectrum of oxidized TMP (notice that the theoretical spectrum cannot account for several absorption bands shown by the experimental spectrum (c) due to the presence in the sample of TMP and other TMP modifications (species illustrated in Table 2)). Some characteristic bands of DMP-CHO, which are relevant for the following discussion, are marked in the figure and the corresponding experimental features are marked in the same way in spectrum (c). They are labelled as α, β, γ in Figure SI-14 and corresponds to the observed strong feature at 1516 cm^{-1}, assigned to C=C stretchings of the ring, to a sharp feature at 1345 cm^{-1} and to the structured band at 1301 cm^{-1} (spectrum c). Dashed lines (spectra c and d) show that these spectral features disappear after the thermal treatment with Anthracene. Moreover, also the CH bending out of plane features below 800 cm^{-1} show a remarkable evolution which cannot be simply ascribed to the occurrence in this region of the strong out-of-plane IR transitions of Anthracene. These observations parallel the predictions based on DFT calculations showing the disappearance of three characteristic features of DMP-CHO after bonding with Anthracene. The analysis of the normal modes eigenvectors associated to these bands confirms that they are due to vibrations involving the CC bonds of the pyrrole ring. These bonds are mostly affected by the formation of the covalent bonds with anthracene, because of the change of the hybridization of the C atoms.
